# Telomere-binding proteins Taz1 and Rap1 regulate DSB repair and suppress gross chromosomal rearrangements in fission yeast

**DOI:** 10.1371/journal.pgen.1008335

**Published:** 2019-08-27

**Authors:** Hiroyuki Irie, Io Yamamoto, Yusuke Tarumoto, Sanki Tashiro, Kurt W. Runge, Fuyuki Ishikawa

**Affiliations:** 1 Department of Gene Mechanisms, Graduate School of Biostudies, Kyoto University, Sakyo-ku, Kyoto, Japan; 2 Department of Molecular Genetics, Cleveland Clinic Lerner College of Medicine, Cleveland, Ohio, United States of America; Netherlands Cancer Institute, NETHERLANDS

## Abstract

Genomic rearrangements (gross chromosomal rearrangements, GCRs) threatens genome integrity and cause cell death or tumor formation. At the terminus of linear chromosomes, a telomere-binding protein complex, called shelterin, ensures chromosome stability by preventing chromosome end-to-end fusions and regulating telomere length homeostasis. As such, shelterin-mediated telomere functions play a pivotal role in suppressing GCR formation. However, it remains unclear whether the shelterin proteins play any direct role in inhibiting GCR at non-telomeric regions. Here, we have established a GCR assay for the first time in fission yeast and measured GCR rates in various mutants. We found that fission yeast cells lacking shelterin components Taz1 or Rap1 (mammalian TRF1/2 or RAP1 homologues, respectively) showed higher GCR rates compared to wild-type, accumulating large chromosome deletions. Genetic dissection of Rap1 revealed that Rap1 contributes to inhibiting GCRs via two independent pathways. The N-terminal BRCT-domain promotes faithful DSB repair, as determined by I-SceI-mediated DSB-induction experiments; moreover, association with Poz1 mediated by the central Poz1-binding domain regulates telomerase accessibility to DSBs, leading to suppression of *de novo* telomere additions. Our data highlight unappreciated functions of the shelterin components Taz1 and Rap1 in maintaining genome stability, specifically by preventing non-telomeric GCRs.

## Author summary

Tips of chromosomes, telomeres, are bound and protected by a telomere-binding protein complex called shelterin. Most previous studies focused on shelterin’s telomere-specific role, and its general role in genome maintenance has not been explored extensively. In this study, we first set up an assay measuring the spontaneous formation rate per cell division of gross chromosomal rearrangements (GCRs) in fission yeast. We found that the rate of GCRs is elevated in mutants defective for shelterin components Taz1 or Rap1. Detailed genetic experiments revealed unexpectedly that Taz1 and Rap1 have a novel role in repairing DNA double-strand breaks (DSBs) and suppressing GCRs at non-telomeric regions. Given that shelterin components are conserved between fission yeast and humans, future studies are warranted to test whether shelterin dysfunction leads to genome-wide GCRs, which are frequently observed in cancers.

## Introduction

The integrity of chromosomal DNA can be compromised by mutations that vary in size, ranging from small perturbations, such as point mutations and short insertions/deletions, to large changes, such as deletions, duplications, inversions, and translocations of long chromosome segments. The latter are collectively called genomic rearrangements or gross chromosomal rearrangements (GCRs), which have profound implications in cancers as well as genetic diseases. Recent advances in DNA sequencing technology have enabled us to trace the history of GCRs in cancer cells, and it is now well-known that cancer development is accompanied by the frequent occurrence of GCRs [1]. Thus, elucidation of the molecular mechanism underlying GCR control is of critical importance in understanding the progression of cancer malignancy.

Previous studies have pointed to the requirement of chromosome maintenance mechanisms for suppression of GCRs, including DNA repair and telomere protection pathways [2, 3]. The telomere is a huge DNA-protein complex that is located at the termini of linear chromosomes. In humans, telomeric DNA comprises hexanucleotide TTAGGG repeats and consists of a double-stranded (ds) region and a single-stranded (ss) overhang. The telomeric dsDNA recruits TRF1-TRF2-Rap1, whereas the ss telomeric DNA recruits POT1-TPP1, and these two subcomplexes are bridged by TIN2 to form a complex known as shelterin (reviewed in [4]). This shelterin complex helps cells distinguish telomeres from DNA double-strand breaks (DSBs) that must be repaired. For instance, TRF2 depletion brings about the frequent occurrence of chromosome end-to-end fusions, which is due to deregulation of the non-homologous end joining (NHEJ) repair pathway at telomeres. Resultant dicentric chromosomes are unstable, leading to another round of chromosomal rearrangements (reviewed in [5]). It is thus evident that telomere protection by the shelterin complex is vital for repressing GCRs.

While the shelterin complex primarily serves to protect telomeric DNA, the telomere-associated DNA polymerase named telomerase is implicated in GCRs [6, 7]. On the one hand, telomerase is able to elongate the telomere repeat sequence using its RNA subunit as a template, thereby counteracting gradual telomere shortening at each round of DNA replication. At the same time, however, telomerase poses a potential threat to genome stability. In budding yeast, telomerase promotes GCRs through *de novo* addition of telomere repeats to DSB sites, resulting in terminal deletion of chromosomal DNA [7]. It has been reported that *de novo* telomere addition is suppressed through two mechanisms: activation of Pif1 helicase, which was proposed to remove telomerase from DSBs; and inhibition of Cdc13 accumulation by DNA damage signaling [8–10]. However, a previous study showed that fission yeast Pif1 is not a negative regulator of telomerase [11]. In human cells, recruitment of telomerase to telomeres and the activity of telomerase are regulated by the shelterin complex (reviewed in [12]). However, it is still unclear whether shelterin is also involved in the regulation of *de novo* telomere addition at non-telomeric sites.

Fission yeast, *Schizosaccharomyces pombe*, serves as a useful model to dissect the functions of shelterin, because this unicellular organism shares most of the shelterin components with humans. Fission yeast shelterin is composed of six proteins: Taz1, Rap1, Poz1, Tpz1, Pot1, and Ccq1. Among these, Taz1, Tpz1 and Pot1 are orthologs of human TRF1/2, TPP1, and POT1, respectively. Fission yeast Rap1 and human Rap1 are also homologous to each other, sharing several domains including a single BRCT domain at their N termini. Taz1 and Rap1 form a subcomplex that binds to telomeric ds DNA, while Poz1, Tpz1, Ccq1, and Pot1 form another subcomplex at the telomeric ss DNA. Similar to human shelterin, these two subcomplexes at telomeric ds and ss DNA are bridged by the physical interaction between Rap1 and Poz1 [13, 14].

To date, the shelterin components in fission yeast have been extensively investigated. Taz1, Rap1, and Poz1 negatively regulate telomerase activity and promote telomere heterochromatin formation [13, 15–17]. On the other hand, Tpz1 and Pot1 are essential for telomere protection, and thus telomere DNA is aggressively degraded after deletion of the *tpz1*^+^ or *pot1*^+^ gene [13, 18]. Ccq1 recruits telomerase to telomeres through direct binding to telomerase [19, 20]. Taz1 and Rap1 prevent telomere end fusions that would otherwise be caused by aberrant activation of the NHEJ repair pathway at telomeres [21, 22]. Taz1 and Rap1 also tether telomeres to the nuclear periphery via inner nuclear membrane (INM) protein Bqt4 in vegetative cell growth [23]. As such, the shelterin components perform distinct functions, even though they form a complex.

It is known that disruption of shelterin can trigger frequent GCRs through breakage of dicentric chromosomes formed by chromosome end-to-end fusion [5]. However, it is unclear whether the shelterin complex has an additional GCR-suppressive function apart from preventing such chromosome end-to-end fusions; this uncertainty can be ascribed to technical limitations in precisely measuring the occurrence rate of GCRs in mammalian cells. In budding yeast, an assay has been developed to measure the GCR rates, aptly termed the “GCR assay” [24, 25]. GCR rates are deduced from loss of two tandem counter-selective markers inserted in a non-essential chromosomal region. In this study, we adopted the GCR assay to fission yeast and examined whether the individual shelterin components as well as other telomere-binding proteins suppress GCRs in non-telomeric regions. We found that a fraction of the shelterin components, including Taz1 and Rap1, are required for GCR suppression. Deletion of DNA ligase IV, which is essential for NHEJ, did not rescue the increased GCR rates in *taz1*Δ and *rap1*Δ mutant cells, suggesting that Taz1 and Rap1 do not prevent GCR via suppressing NHEJ, unlike the Taz1- and Rap1-dependent protection of telomeres from fusion. Instead, derepression of telomerase is responsible for the increased GCR rates in *taz1*Δ and *rap1*Δ strains. Dissection of the Rap1 protein identified the N-terminal BRCT domain as an important domain for the GCR suppression. Moreover, when DSBs are site-specifically induced at a non-telomeric locus by I-SceI endonuclease, Taz1 and Rap1 are required for cellular survival and for inhibiting erroneous repair. We propose that Taz1 and Rap1 prevent GCRs by regulating telomerase activity and DSB repair, even in non-telomeric regions.

## Results

### Measurement of spontaneous GCR rates in fission yeast cells

**Fig 1:**
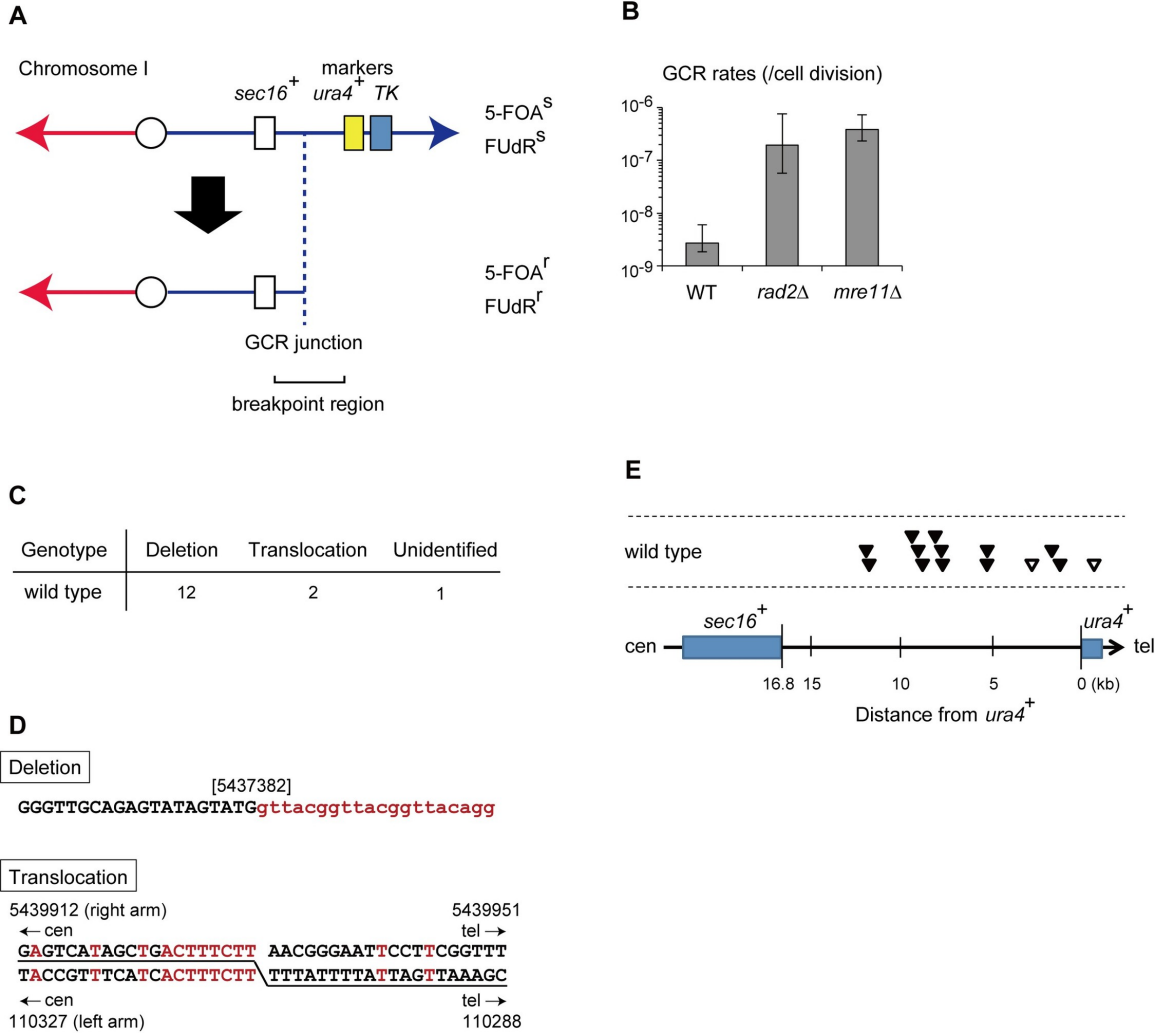
Measurement of spontaneous GCR rates in fission yeast. (A) Schematic structure of the *ura4*-*TK* strain for the GCR assay. *ura4*^+^ and *TK*, which confer sensitivity to 5-fluoroorotic acid (5-FOA) and 5-fluoro-2'-deoxyuridine (FUdR), respectively, were inserted in the right arm of chromosome I. *sec16*^+^ is the most distal essential gene on the right arm. (B) GCR rates, which are median rates for the accumulation of 5-FOA/FUdR-resistant progeny, are plotted on a log scale for each strain. In all the figures showing GCR rates in this report, error bars represent the >95% confidence intervals. (C) Numbers of different types of GCRs identified in 5-FOA/FUdR-resistant clones derived from wild-type strain. (D) Representative breakpoint junction sequences in GCR survivors derived from wild-type cells. In the case of a deletion type survivor (upper panel), telomeric repeats are indicated by lowercase letters in red. The number in brackets indicates the nucleotide coordinate of the chromosome I at which the telomere repeats were added. In the case of a translocation type survivor (lower panel), the upper and lower sequences are the original chromosomal DNA sequences proximal to the translocation, and the underlined nucleotides indicate the fused sequence. The red letters indicate identity. The reference sequence was obtained from the Pombase website (http://www.pombase.org/). See S1B Fig and S2 Table for the detailed sequential changes. (E) Distribution of GCR junctions in the breakpoint region. Each arrowhead represents the location of a junction in the 5-FOA/FUdR-resistant clones. Closed and open arrowheads indicate deletion and translocation types, respectively.

To measure GCR rates in fission yeast, we applied the assay system that was previously developed for budding yeast (Fig 1A) [24]. We constructed a DNA cassette containing two neighboring marker genes, *ura4*^+^ and *TK* (the latter encodes herpes virus thymidine kinase) in tandem. Cells expressing *ura4*^+^ and *TK* are sensitive to 5-fluoroorotic acid (5-FOA) and 5-fluoro-2’-deoxyuridine (FUdR), respectively. As expected, fission yeast cells with this marker cassette integrated at approximately 150 kb from the right telomere of chromosome I (the precise location is described in Materials and Methods) showed sensitivity to both of the drugs (5-FOA/FUdR) (S1A Fig). This strain is expected to become resistant to both drugs when the *ura4*^+^ and *TK* genes undergo simultaneous deletions and/or loss-of-function point mutations. However, such simultaneous point mutations seem highly unlikely because the probability of simultaneous point mutations occurring in two specific genes is thought to be quite low (~10^−14^/cell division, given that the spontaneous incidence of loss-of-function mutations for each gene, independently, is ~10^−7^, see Methods) [26]. Thus, as in the budding yeast GCR assay system, the vast majority of drug-resistant survivors in our assay should be derived from GCRs that result in simultaneous loss of the two marker genes. Because an essential gene closest to the marker cassette is *sec16*^+^, which is located about 16.8 kb centromeric from the cassette, and there is no essential gene telomeric to the cassette, our system can detect GCRs that take place within this ~16.8-kb region (Fig 1A). Hereafter, we will refer to this GCR target region as the “breakpoint region”. Because it lacks any sequence that shares apparent homology with other chromosome regions, our GCR assay is expected to detect GCRs that are mediated by no or little homology.

In the GCR assay, we counted the number of colonies on a plate with or without 5-FOA/FUdR and estimated GCR rates per cell division using fluctuation analysis [24]. In the case of wild-type cells, a GCR rate determined in our system was 2.6 × 10^−9^ per cell division (Fig 1B). This rate is actually far greater than the expected probability of dual independent point mutant survivors (10^−14^ per cell division), confirming that our system primarily detects GCRs. We then isolated 5-FOA/FUdR-resistant clones and performed DNA sequencing at the breakpoint region (See Materials and Methods). Based on the sequencing data, GCRs were classified into deletion and translocation types (Fig 1C, wild-type). In the deletion type, DSBs led to deletion of the chromosomal terminus containing the drug selection cassette. The sequencing analysis detected ectopic telomeric DNA repeats at the breakpoints, suggesting that *de novo* telomere addition healed the DSB (Fig 1D). Twelve out of fifteen wild-type-derived GCR clones examined here belonged to this type. In two other clones, breakpoints were fused with unique sequences from the left arm of chromosome I (opposite the right arm where the original marker cassette had been located prior to the rearrangement) in a head-to-tail orientation (same direction towards telomeres). Such fusions could be derived from either break-induced DNA replication or DNA recombination (Fig 1D, Translocation as diagramed in S1C Fig). Indeed, the breakpoint junctions consisted of 7 or 8 bp of microhomology in these survivors (Fig 1D). In both types of GCRs, the locations of the breakpoints seemed to be uniformly distributed in the breakpoint region, rather than clustering at a particular hotspot (Fig 1E). In the last of the 15 survivors, we established the loss of the marker cassette but could not determine the precise change in the breakpoint region sequence. As we expected, we did not obtain any clones with simultaneous point mutations in both *ura4*^+^ and *TK*, validating the usefulness of our assay system to specifically evaluate GCR rates.

Of note, the GCR rate measured in our fission yeast system is comparable to that in the original budding yeast system: 2.6 × 10^−9^ /cell division in a 16.8 kb-long breakpoint region in wild-type fission yeast (this study) vs. 2.27 × 10^−9^ in a 19.2 kb breakpoint region in wild-type budding yeast [27]. If we assume that GCRs occur randomly throughout the genome, then GCR rates would be expected to be proportional to the DNA length of the breakpoint region for the GCR assay. The GCR rates normalized to unit length are 1.5 × 10^−10^/cell division/kb in fission yeast and 1.2 × 10^−10^/cell division/kb in budding yeast. Thus, wild-type cells of the two yeast species showed comparable normalized GCR rates. In addition, GCRs in wild-type fission yeast cells are mostly associated with terminal deletions, whereas translocations are relatively rare, just as in budding yeast [24]. We thus asked whether the GCR suppression mechanism identified by the budding yeast GCR assay system also functions in fission yeast. Budding yeast strains lacking nuclease FEN-1 or Mre11 show a 914- and 628-fold increases in GCR rates, respectively [20]. We therefore tested the effects of loss of FEN-1 and Mre11 in *rad2Δ* and *mre11Δ S*. *pombe* cells and observed a ~100-fold increase in GCR rates (Fig 1B). In budding yeast, Pif1 helicase suppresses telomerase-mediated telomere elongation at native telomeres and DSBs through destabilizing annealing of telomerase RNA template and single-stranded telomere DNA substrates [28]. We examined the impact of inactivating *pfh1*, the fission yeast homologue of *PIF1* on GCRs. pfh1-mt* is a mutant that lacks nuclear functions but retains the essential mitochondrial functions [29]. pfh1-mt* showed 32-fold higher GCR rates than wild type, similar to the results reported for the budding yeast corresponding mutant, *pif1-m2* (S1D Fig) [7]. From these similarities observed in the two distinct yeast species, we surmise that the regulatory mechanism suppressing GCRs is evolutionarily conserved, underscoring the significance of studying the GCR mechanism in fission yeast.

### Taz1 and Rap1 suppress GCRs

**Fig 2:**
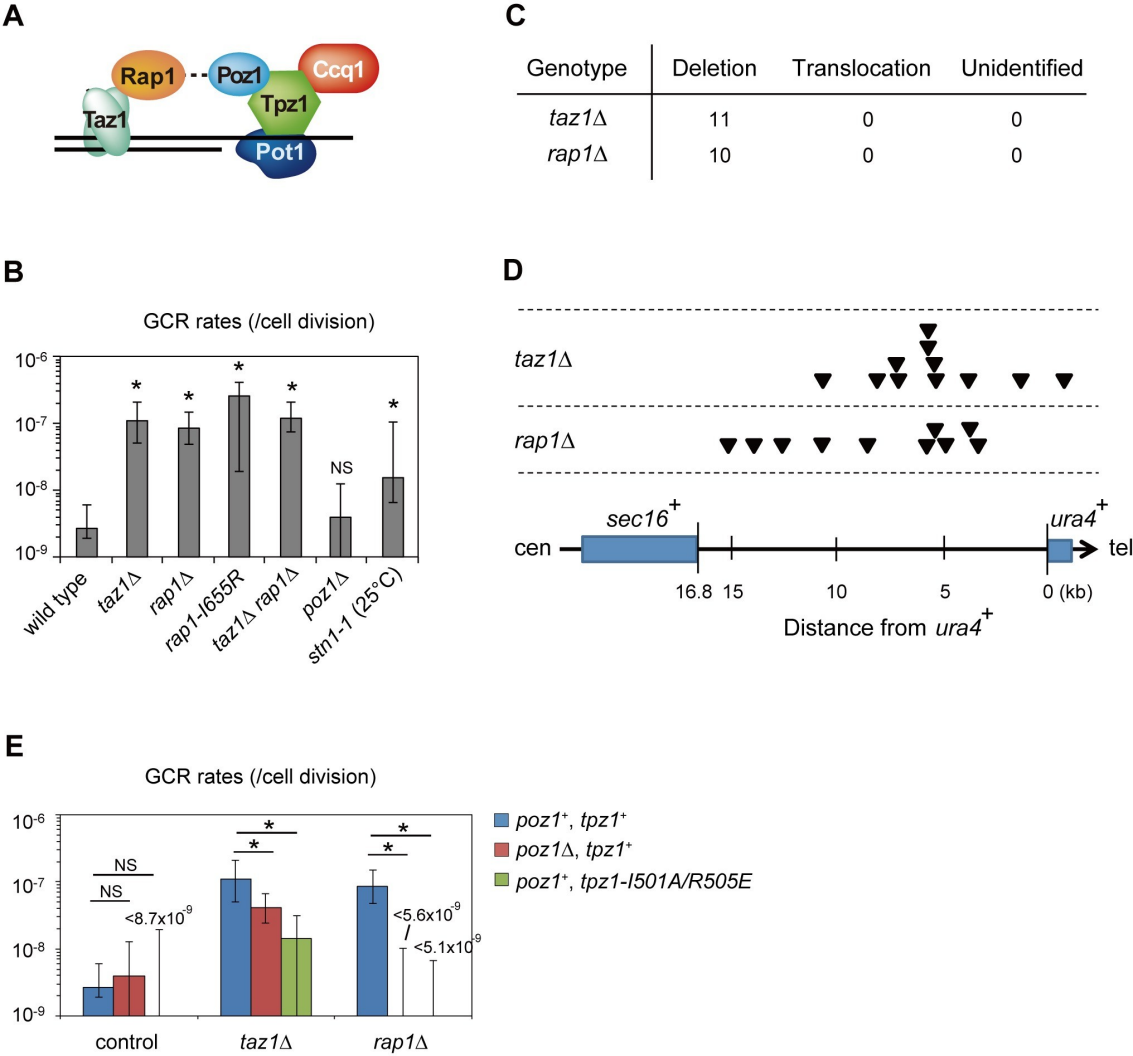
Regulation of GCRs by individual telomere binding proteins. (A) Schematic representation of the *S*. *pombe* shelterin complex. (B) GCR rates of telomere-binding protein mutants. (C) Numbers of different types of GCRs identified in 5-FOA/FUdR-resistant clones derived from strains with the indicated genetic backgrounds. (D) Distribution of GCR (exclusively deletion types) junctions in the breakpoint region. Each arrowhead represents the location of a junction in the 5-FOA/FUdR-resistant clones. (E) GCR rates of *poz1*Δ and *tpz1-I501A/R505E* mutants in wild-type, *taz1*Δ, and *rap1*Δ backgrounds. Asterisks represent a statistically significant difference (P < 0.05) from the wild-type strain, unless otherwise noted. NS: non-significant (P > 0.05). P-values were determined by the two-tailed Mann–Whitney test.

Since most of the GCR survivors that we isolated were derived from *de novo* telomere addition, we investigated a possible involvement of the telomere proteins in GCR regulation. In fission yeast, Taz1 directly binds to both Rap1 and ds telomeric DNA, thereby recruiting Rap1 to telomeres (Fig 2A). We found that *taz1*Δ and *rap1*Δ cells showed greatly increased GCR rates, 1.1 × 10^−7^ and 0.85 × 10^−7^ /cell division (42-fold and 33-fold higher than wild-type cells), respectively (Fig 2B). A Rap1-I655R mutant, in which the recruitment of Rap1 to telomeres is diminished due to a compromised Taz1-Rap1 interaction [30], showed an increased GCR rate that was comparable to *taz1*Δ or *rap1*Δ (Fig 2B, 2.6 × 10^−7^ /cell division, a 97-fold increase over wild-type), suggesting that Taz1 represses GCRs primarily through the physical interaction with Rap1. Consistent with this notion, *taz1*^+^ and *rap1*^+^ were found to be epistatic: a *taz1*Δ *rap1*Δ double mutant (1.2 × 10^−7^ /cell division) showed similarly increased GCR rates compared to each single mutant *taz1*Δ or *rap1*Δ (Fig 2B). We determined the sequences of GCR breakpoints in *taz1*Δ and *rap1*Δ survivors in GCR assay. We found only deletion type GCRs in *taz1*Δ and *rap1*Δ survivors, although the fractions of deletion in these mutants are not significantly higher than in wild type (Fig 2C & 2D). These results imply that the two shelterin components Taz1 and Rap1 function in the same pathway to prevent GCRs.

We also measured GCR rates of wild-type, *taz1*Δ, and *rap1*Δ strains at 20°C. It is known that *taz1*Δ, but not wild-type or *rap1*Δ delay the cell cycle progression and lose viability due to chromosomal entanglement at this temperature [31]. We found that *taz1*Δ, but not wild-type and *rap1*Δ, showed more than one order of magnitude higher GCR rates at 20°C than at 32°C (S2 Fig). Given the correlation between the increased GCR rate and cold-sensitivity among the three strains, it is possible that the chromosome entanglement contributes to the high GCR rate with *taz1*Δ at 20°C. Future study is necessary for concluding the molecular link between these phenotypes.

In sharp contrast to *taz1*^+^ *and rap1*^+^ deletion, deletion of the *poz1*^+^ gene, which encodes another Rap1-interacting shelterin component, did not affect the GCR rate (3.9 × 10^−9^ /cell division, Fig 2B). Interestingly, *taz1*Δ *poz1*Δ and *rap1*Δ *poz1*Δ double mutants showed lower GCR rates than the *taz1*Δ and *rap1*Δ single mutants, demonstrating that Poz1 is required for the derepression of GCRs in *taz1*Δ and *rap1*Δ cells (Fig 2E). Consistently, the abrogation of Poz1-Tpz1 binding by a *I501A/R505E* mutation in Tpz1 [32], another shelterin component that directly binds to Poz1, similarly suppressed the increased GCR rates in *taz1*Δ and *rap1*Δ mutant backgrounds (Fig 2E). This result suggests that Poz1 recruitment promotes GCRs in *taz1*Δ and *rap1*Δ, given that Poz1-Tpz1 binding is essential for telomere localization and function of Poz1 [14, 33]. We noticed that *poz1*^+^ deletion and Tpz1-I501A/R505E mutation individually caused strong reduction in GCR rates in *rap1*Δ but not much in *taz1*Δ cells. These results suggest that the increased GCR rates in *taz1*Δ and *rap1*Δ are caused by different mechanisms. It was reported that the formation of Rap1-Poz1-Tpz1 trimer is a hierarchical process *in vitro* [34]. First, Poz1 and Tpz1 form a dimer. The Rap1-binding domain of Poz1 undergoes allosteric changes upon the Poz1-Tpz1 dimer formation, which greatly increases the affinity with Rap1, and induces the Rap1-Poz1-Tpz1 trimer formation. Therefore, it is possible that the Tpz-Poz1 dimer stably exists in *rap1*Δ but not in *taz1*Δ. Such unusual shelterin subcomplexes may contribute to the differential effects of *poz1*^+^ deletion in *taz1*Δ and *rap1*Δ, as revealed in Fig 2E. These results suggest that genetic interaction of Taz1, Rap1, and Poz1 regarding GCR suppression is complex, similarly to that as for telomere length regulation and cold sensitivity [20, 22]. We also examined Stn1, a non-shelterin protein that binds to telomeric ssDNA. Because Stn1 is essential for telomere protection, we investigated a temperature sensitive mutant *stn1-1*, which has slightly elongated telomeres at semi-permissive temperature 25°C [35]. We found a moderately increased GCR rate at that temperature (Fig 2B), 1.6 × 10^−8^ /cell division, a 6-fold increase over wild-type.

### NHEJ is not essential for increased GCR rates in *taz1*Δ and *rap1*Δ

**Fig 3:**
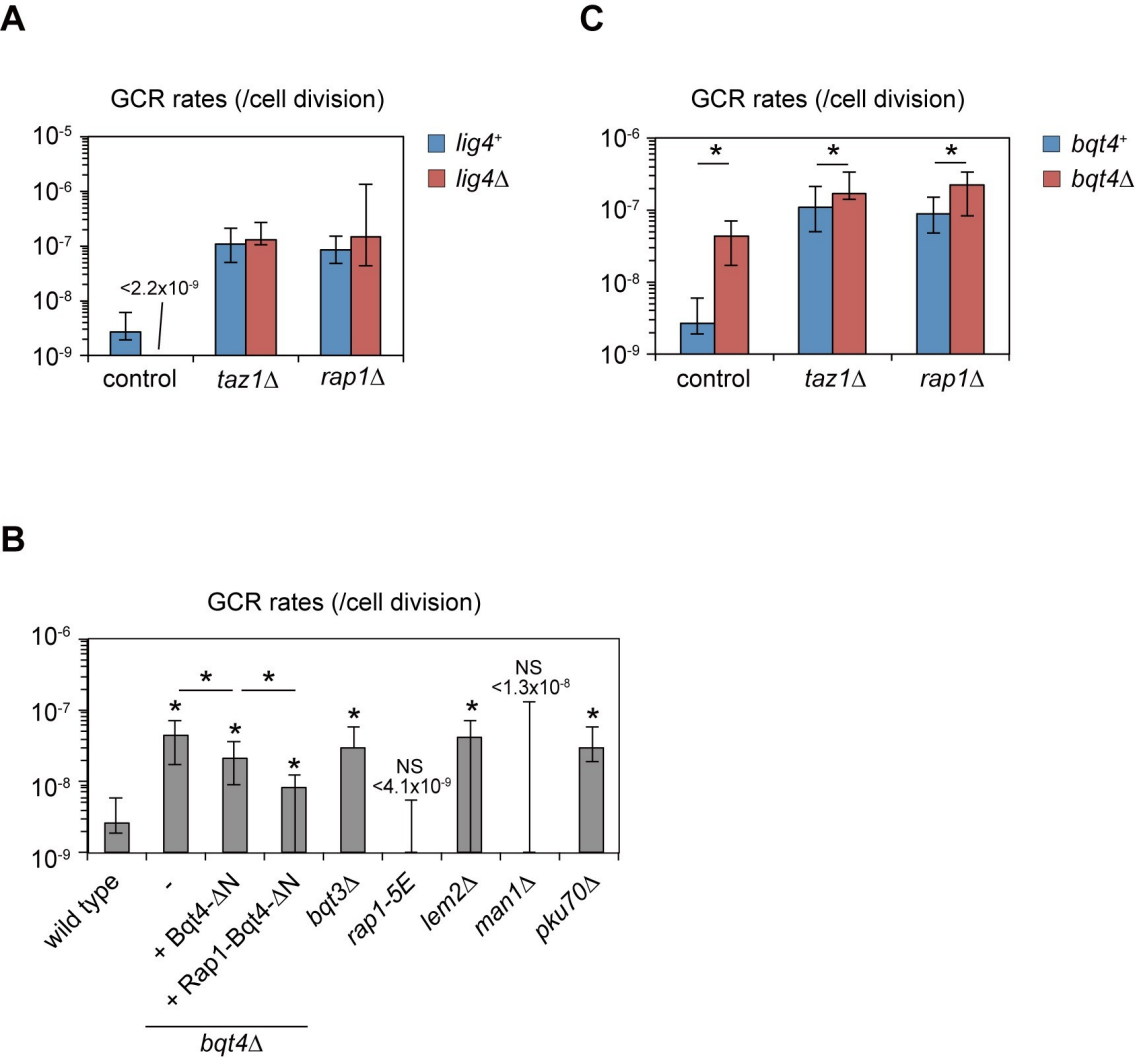
Effects of telomere end-to-end fusion and telomere tethering to the nuclear envelope on GCR formation. (A) GCR rates of wild-type, *taz1*Δ, and *rap1*Δ strains in the presence or absence of *lig4*^+^. (B) GCR rates of strains with INM protein mutants. GFP-Bqt4ΔN or Rap1-GFP-Bqt4ΔN fusion protein was expressed from the *bqt4* promoter integrated into genomic DNA as described previously [23]. (C) GCR rates of wild-type, *taz1*Δ, and *rap1*Δ strains in the presence or absence of *bqt4*^+^. Asterisks represent significant differences (P < 0.05) from the wild-type strain unless otherwise noted. P-values were obtained by the two-tailed Mann–Whitney test.

Because both Taz1 and Rap1 are multi-functional (see below), we set out to dissect which specific function(s) is related to the GCR inhibition. It is known that, in *taz1*Δ and *rap1*Δ cells, but not in *poz1*Δ cells, telomeres are prone to fuse to each other by NHEJ when cells are arrested at G1 phase [21, 36]. It is thus possible that a failure to suppress NHEJ in *taz1*Δ and *rap1*Δ could lead to formation of dicentric chromosomes, which would trigger DSBs which could result in the observed chromosome terminal deletions. In order to examine whether NHEJ is responsible for frequent GCRs in *taz1*Δ and *rap1*Δ mutant cells, we deleted the DNA ligase IV-encoding *lig4*^+^gene, which is essential for NHEJ in fission yeast. It was reported that a lack of *lig4*^+^ suppresses the frequent telomere fusions in *taz1*Δ and *rap1*Δ [21, 22]. We found that disruption of *lig4*^+^ in *taz1*Δ and *rap1*Δ did not significantly suppress the increased GCR rates observed with *taz1*Δ and *rap1*Δ (Fig 3A), suggesting that NHEJ is dispensable for the high incidence of GCRs in *taz1*Δ and *rap1*Δ cells.

Because fission yeast in exponentially growing phase shows very short G1 phase, and NHEJ is active only in G1 but not in S and G2 phase, it was possible that NHEJ was dispensable for GCRs due to a small fraction of cells staying in G1 phase. We therefore arrested cells in G1 phase through nitrogen starvation, and measured the GCR frequency. Briefly, cells exponentially growing in YES media were divided into two groups, which were incubated in EMM media with (N+) or without (N-) ammonium sulfate for 24 hr, respectively, and then transferred to YES media for growth overnight. Then equal numbers of N+ and N- cells were subjected to the GCR assay. We found that *taz1Δ*(N-) cells showed an approximately two-fold increase in GCR frequencies compared to *taz1Δ*(N+) cells (S3A Fig). Because *taz1*Δ(N-) cells were G1-arrested and/or lost viability [21] while *taz1*Δ(N+) cells actively proliferated in EMM media (with or without supplementing nitrogen), the total number of cell divisions was greater in *taz1*Δ(N+) cells than in *taz1*Δ(N-) cells. Therefore, the two-fold increase of GCR frequencies is most likely an underestimate of a larger GCR rate (which is normalized per cell division) in *taz1*Δ(N-) compared to that in *taz1*Δ(N+). Interestingly, GCR frequencies in *taz1*Δ(N-) were partially suppressed by ligase IV deletion, while those in *taz1*Δ(N+) were not. Taken together, NHEJ also contributes to the increase of GCR frequencies of *taz1*Δ cells in G1 phase. Because G1 cells are rare in cells with unperturbed cell cycles, this effect is negligible in exponentially growing cell populations. These results suggest that the break-fusion-bridge cycle via formation of telomere-fusion-mediated dicentric chromosomes plays a minor role, if any, in the increased GCR rate in cycling *taz1*Δ cells.

### Heterochromatin plays a minor role in controlling GCRs in *rap1*Δ

Both Taz1 and Rap1 are essential for heterochromatin formation at telomeres and their adjacent regions, subtelomeres [37]. To examine whether telomere heterochromatin structure is important for suppression of GCRs, we deleted the *clr4*^+^ and *swi6*^+^ genes, both of which encode essential factors for heterochromatin formation (S3B Fig). Deletion of *swi6*^+^ in the wild-type background led to a small increase in the GCR rate, suggesting a potential contribution of heterochromatin to the suppression of GCRs. We examined *poz1-W209A* mutation. The shelterin component Poz1 is required for telomere silencing, and the *poz1-W209A* mutation is known to specifically disrupt the telomere heterochromatin regulatory function, among others [38]. No increase in the GCR rate was observed in *poz1-W209A* strain (S3C Fig). In contrast, deletion of *clr4*^+^ in *rap1*Δ background showed small but significant decrease GCR rates, although the underlying mechanism is unclear. From these results, we conclude that heterochromatin does not play a significant role in suppressing GCR except in *rap1*Δ.

### Inner nuclear membrane proteins, including Bqt4, suppress GCRs, but are not epistatic with Taz1 and Rap1

In fission yeast, Taz1 and Rap1, but not Poz1, tether telomeres to the INM via binding of Rap1 to INM protein Bqt4 in vegetative cell growth [23, 39]. It is thus possible that the telomere tethering to the INM contributes to GCR suppression through regulation of chromosome positioning within the nucleus. We found that *bqt4*Δ cells showed moderately increased GCR rates (4.3 × 10^−8^ /cell division, Fig 3B). The GCR rate was also significantly increased by deletion of *bqt3*^+^ (3.0 × 10^−8^ /cell division), whose protein product Bqt3 stabilizes Bqt4 [23]. It was reported that the Ku70/80 complex and two INM proteins Lem2 and Man1 also promote tethering of telomeres to the nuclear envelope, although Man1 plays a minor role [40, 41]. Deletion of *pku70*^+^ or *lem2*^+^, but not *man1*^+^, led to moderately higher GCR rates (2.9 × 10^−8^ and 4.2 × 10^−8^ /cell division, respectively) than the wild-type strain (Fig 3B). These results imply that tethering of telomeres to the nuclear envelope facilitates GCR suppression.

Bqt4 localizes to the INM through its C-terminus transmembrane domain, and its N-terminal half is necessary and sufficient for binding Rap1 [23]. While telomeres are dissociated from the nuclear envelope in *bqt4*Δ, expression of an artificial fusion protein between Rap1 and an N terminus-truncated Bqt4 (Rap1-GFP-Bqt4ΔN) in *bqt4*Δ resumed telomere clustering at the nuclear envelope [23]. With our GCR assay, we found that *bqt4*Δ cells expressing the Rap1-GFP-Bqt4ΔN fusion protein from *bqt4* promoter showed only a slightly lower GCR rate than *bqt4*Δ cells expressing GFP-Bqt4ΔN, in which telomeres are not tethered to the INM (Fig 3B, 2-fold difference). Moreover, the *rap1-5E* mutant (consisting of S213E, T378E, S422E, S456E, S513E mutations), in which the interaction between Rap1 and Bqt4 is impaired [39], displayed a comparable GCR rate to wild-type cells (Fig 3B). We also found that simultaneous deletion of *bqt4*^+^ significantly increased GCR rates in *taz1*Δ and *rap1*Δ cells (Fig 3C). These results suggest that Rap1-Bqt4 binding plays a minor role in suppressing GCRs, and that Bqt4 regulates GCRs at least in part by a Taz1- and Rap1-independent mechanism. By the same token, this result suggests that Rap1 utilizes Bqt4-independent mechanisms for suppressing GCRs.

### Deregulated telomerase activity is essential for the increase in GCRs in *taz1*Δ and *rap1*Δ cells

**Fig 4:**
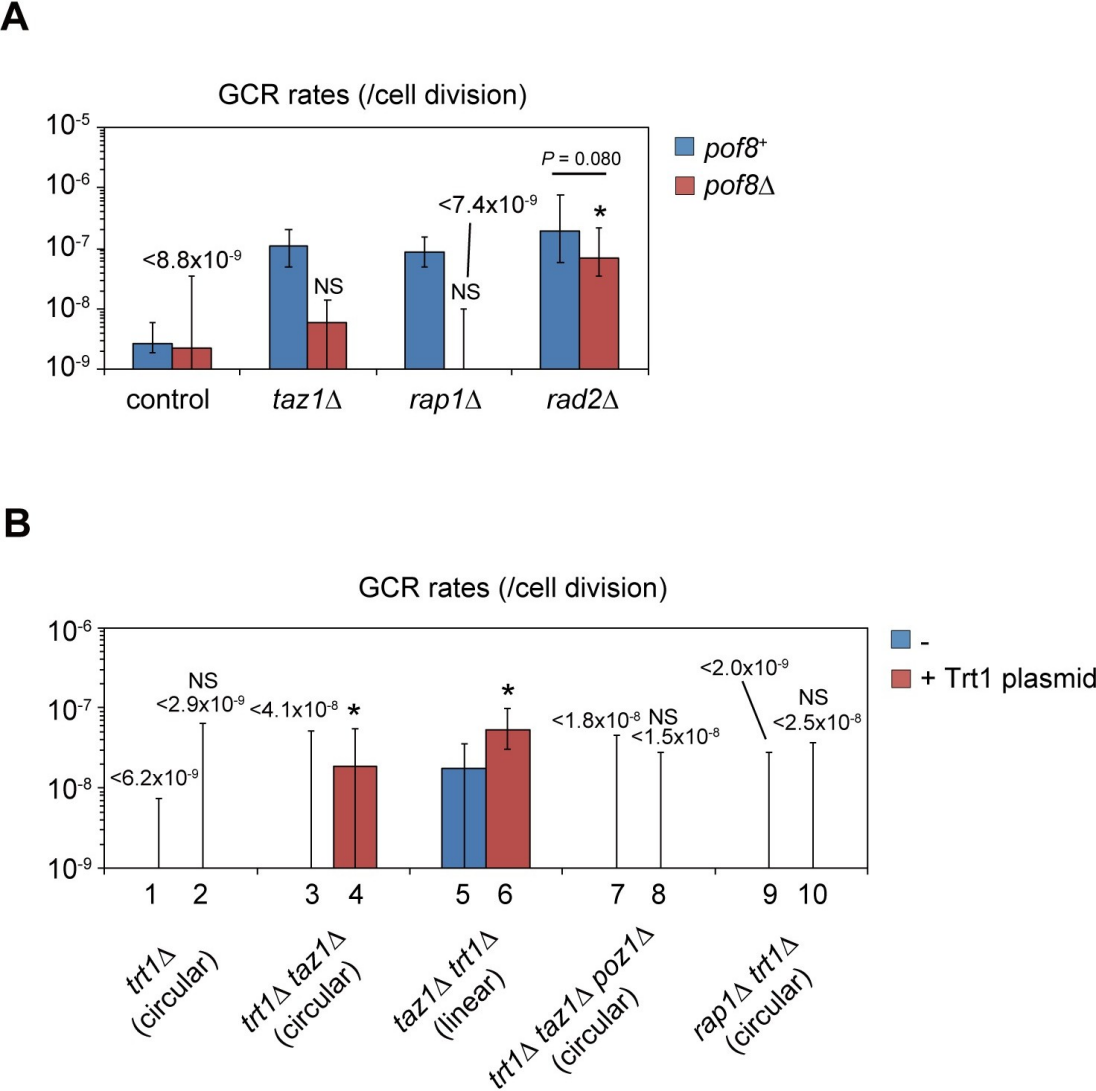
Telomerase activity is essential for frequent GCRs in *taz1*Δ and *rap1*Δ strains. (A) GCR rates of wild-type, *taz1*Δ, *rap1*Δ, and *rad2*Δ strains in the presence or absence of *pof8*^+^. (B) GCR rates of *trt1*Δ strains in the presence or absence of a pREP1-*trt1*^+^ plasmid (Trt1 plasmid). The GCR assay was performed in YES media, in which the *nmt1* promoter in the plasmid has a moderate activity [69]. Asterisks and NS represent significant differences (P < 0.05 and P>0.05) from the wild type strain, respectively. P-values were obtained by the two-tailed Mann–Whitney test.

Taz1 and Rap1 suppress telomerase-mediated telomere DNA elongation [42, 43]. Given that all GCRs examined in *taz1*Δ and *rap1*Δ were terminal deletions involving *de novo* telomere additions at breakpoints, it was likely that deregulated telomerase reactions facilitated GCRs through enhanced *de novo* telomere addition in *taz1*Δ and *rap1*Δ cells. Inactivation of *trt1*^+^, the gene encoding the catalytic subunit of telomerase, leads to chromosome self-circularization [42], making the GCR assay results difficult to compare with other cases. We therefore explored the effect of a Pof8 disruption on GCR rates. Pof8 is involved in maturation of telomerase RNA, and deletion of *pof8*^+^ leads to telomere shortening without extensive chromosome circularization, in contrast to a *trt1*^+^ deletion [44–47]. We found that *taz1*Δ *pof8*Δ and *rap1*Δ *pof8*Δ cells, which also do not show chromosome circularization, showed GCR rates which were lower than *taz1*Δ and *rap1*Δ cells, and similar to wild type cells (Fig 4A). These results suggest that telomerase activity is essential for the high GCR rates in *taz1*Δ and *rap1*Δ. In contrast, the GCR rate of a *rad2*Δ *pof8*Δ strain was in between that of *rad2Δ* alone and wild type cells, suggesting that Taz1 and Rap1 specifically suppress telomerase-dependent GCRs.

We considered two possibilities for how telomerase activity affects GCRs in *taz1*Δ and *rap1*Δ: (1) increased telomerase accessibility directly facilitates *de novo* telomere addition at breakpoints, or (2) abnormally elongated native telomeres indirectly affect non-telomeric GCRs. To determine which is the case, we examined GCR rates using cells with circular chromosomes in the presence or absence of Trt1 [4]. It is known that circular chromosomes in *trt1*Δ do not contain telomere DNA sequences [48]. For this purpose, we introduced a Trt1-expressing plasmid into *trt1*Δ cells with circularized chromosomes. In this setting, the majority of the *trt1*Δ cells harboring the Trt1 plasmid maintained circular chromosomes I and II (S4 Fig). As for *trt1*Δ *taz1*Δ, chromosomal configuration depends on the order of gene deletions during the strain preparation. When *trt1*^+^ is deleted first, followed by *taz1*^+^ deletion, the strain contains circular chromosomes. In contrast, linear chromosomes are maintained when *taz1*^+^ is deleted first, followed by *trt1*^+^ deletion [42]. Below, we will describe experiments using *trt1*Δ *taz1*Δ maintaining circular chromosomes, except otherwise noted. We also confirmed that *trt1*Δ *taz1*Δ expressing ectopic Trt1 retains circular chromosomes (S4 Fig).

When we subjected circular chromosome-containing cells to the GCR assay, it was expected that circular chromosomes needed to undergo complicated changes, such as two independent DSBs at the both sides of the selection cassette, and healing of the two DSBs by telomere addition to produce linear chromosomes. Consistently, all of the various strains maintaining circular chromosomes (except Trt1-overproducing *trt1*Δ *taz1*Δ) showed GCR rates below the detection sensitivity of the assay (Fig 4B).

When *trt1*Δ, *trt1*Δ *taz1*Δ and *trt1*Δ *rap1*Δ (all containing circular chromosomes) were transformed with Trt1-expressing plasmids, *trt1*Δ *taz1*Δ showed a significant increase in GCR frequency, while *trt1*Δ and *trt1*Δ *rap1*Δ did not (Fig 4B). These results suggest two points: first, Taz1 prevents GCR formation independent of its specific DNA binding to telomere DNAs, since circular chromosomes lack all telomere DNAs [48]; second, Taz1 has additional roles, which are not shared by Rap1, in preventing GCR formation from circular chromosomes. When we deleted *poz1*^+^ in *trt1*Δ *taz1*Δ cells, followed by over-expression of Trt1, GCR rates were decreased, suggesting that Poz1 promotes GCRs in *taz1*Δ cells in the absence of telomere DNA (Fig 4B, compare lanes 4 and 8). *rap1*Δ *trt1*Δ cells showed similar GCR rates to wild type even after Trt1 re-expression. We confirmed that both *trt1*Δ *taz1*Δ *poz1*Δ cells and *rap1*Δ *trt1*Δ cells maintained circularization of chromosomes I and II before and after Trt1 re-expression (S4 Fig).

In contrast to circular chromosomes-containing *trt1*Δ *taz1*Δ, linear chromosome-maintaining *trt1*Δ *taz1*Δ (see above), showed significantly increased GCR rates compared to linear-chromosome-containing wild-type cells (Fig 4B). Ectopic Trt1-over-expression further increased the GCR rates to the level of *taz1*Δ cells.

### The Rap1 BRCT domain is important for suppression of GCRs

**Fig 5:**
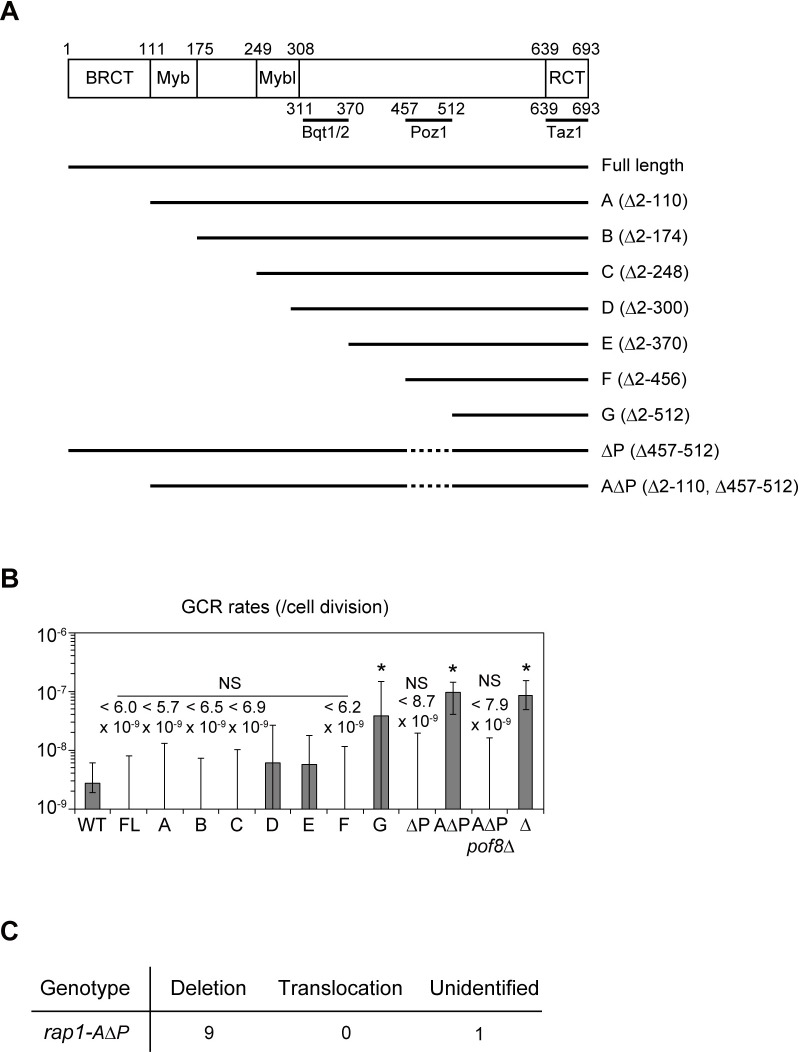
The Poz1-binding domain and BRCT domain of Rap1 redundantly suppress GCRs. (A) Schematic of Rap1 truncation mutants. (B) GCR rates of the Rap1 mutants shown in (A). Rap1 proteins were expressed from a single copy of cognate alleles integrated downstream of the native *rap1* promoter. FL, full length. Asterisks represent significant differences (P < 0.05) from the wild type strain. P-values were obtained by the two-tailed Mann–Whitney test. (C) Numbers of different types of GCRs identified in 5-FOA/FUdR-resistant clones derived from wild-type strain.

To further dissect the precise mechanism of GCR repression by Rap1, we exploited previously reported sequential N-terminal Rap1 truncations, Rap1-A to G [31] (Fig 5A). Among these, we found that only the Rap1-G mutant showed an increased GCR rate. Because the Rap1-A to F mutant strains all retain the Poz1-binding domain (Rap1 457–512 amino acids) but Rap1-G does not, the results raised the possibility that Rap1-Poz1 binding is required for the GCR suppression. However, deletion of the Poz1-binding domain alone did not increase GCR rates (Rap1ΔP, Fig 5A and 5B). Further dissection of Rap1 revealed that simultaneous deletion of the BRCT domain at the N terminus as well as the Poz1-binding domain led to an increase in GCR rates that was comparable to that in *rap1*Δ cells (Rap1-AΔP, Fig 5A and 5B, and S4 Fig). The phenotype of the *rap1-AΔP* strain was similar to *rap1*Δ regarding GCR suppression; GCRs from the *rap1-AΔP* strain primarily showed terminal deletion (Fig 5C), and deletion of *pof8*^+^ canceled the increased GCR rates. These results indicate that the BRCT domain and the Poz1-binding domain redundantly suppress GCRs. Since the Rap1-A mutant, which lacks the BRCT domain, maintains normal telomere length, we suggest that the BRCT domain does not regulate telomerase action at native telomeres, while the Poz1-binding domain suppresses GCRs through inhibition of telomerase at both telomeres and non-telomeric DSBs. The Rap1 BRCT domain may be involved in a general DNA repair pathway, failure of which causes various consequences including erroneous telomere addition by telomerase at non-telomeric regions.

### Taz1-Rap1 promotes cell survival and suppresses terminal deletion in response to an I-SceI induced DSB

**Fig 6:**
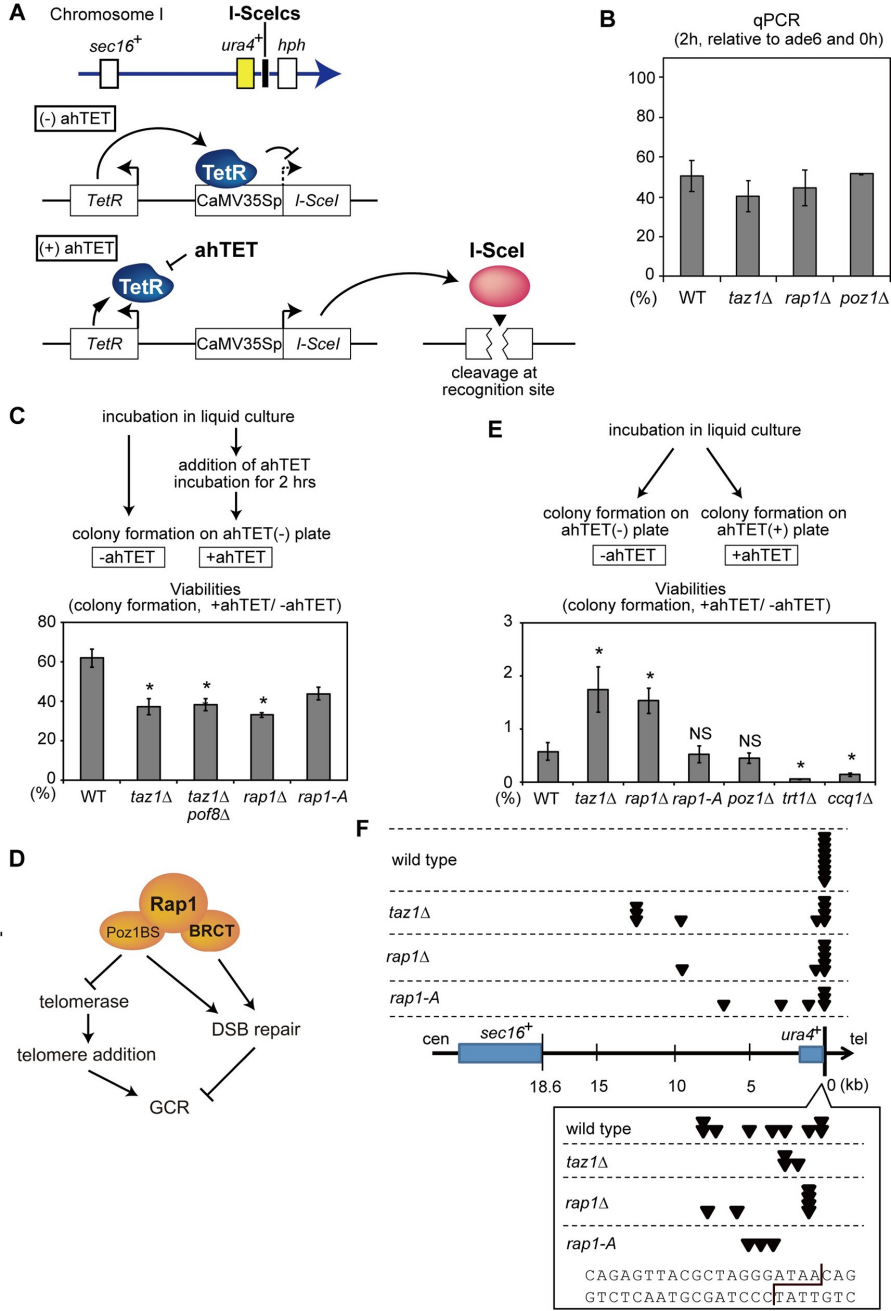
Taz1 and Rap1 are involved in the repair of I-SceI-induced DSBs. (A) Schematic of our site-specific DSB induction system. A gene that encodes endonuclease I-SceI was placed under a modified CaMV 35S promoter that can be repressed by the Tet repressor (TetR) [67]. I-SceIcs was inserted at the same site as the marker gene cassette in the *ura4-TK* strain. A DSB at I-SceIcs can be induced by TetR inhibition via ahTET treatment. (B) Efficiency of DSB induction in I-SceIcs. At the indicated time points after addition of ahTET, DNA breaks were monitored by qPCR with a primer set flanking I-SceIcs. (C) Frequencies of colony-forming cells on non-selective agar plates with or without ahTET treatment for 2 hours. Asterisks represent significant differences (P < 0.05) from the wild-type strain. P-values were obtained by the two-tailed Student’s t-test. We found that I-SceI digestion proceeded for additional two hours ahTET washout (S6A Fig), likely due to residual I-SceI activity remaining after the expression shut-down. Therefore, the digestion efficiencies were likely even greater than the 40~50% indicated in (B). (D) Current model of how Rap1 functions in DSB repair and GCR suppression. See text for details. (E) Survival rates determined by counting numbers of colonies formed on ahTET-containing and non-selective agar plates. (F) Locations of the GCR junctions in the survivors isolated in (E). All survivors were examined for GCR structures as in Fig 1C and 1E. All clones had chromosomal terminal deletions, and none of them displayed translocations. We could not identify the GCR type and location of the junction in one out of the ten examined survivors in both the WT and *taz1*Δ strains. Each triangle (arrowhead) represents the location of the GCR junctions. The inset shows the sequences around I-SceIcs. The cleavage pattern of I-SceI is shown with a line. Regarding (B), (C), and (E), mean values ± SEM are shown. N = 3.

Given that GCRs are thought to arise from aberrant DSB repair, the telomerase-independent GCR repression mechanism could potentially include DSB processing. In order to examine this possibility, we constructed a conditional, site-specific DSB induction system (Fig 6A). The DNA sequence-specific endonuclease I-SceI was expressed under the control of a tetracycline-inducible promoter, and a single I-SceI cut site (I-SceIcs) was integrated at approximately 150 kb centromeric from the right telomere of chromosome I, exactly at the same locus as the marker cassette that was inserted in our GCR assay strains. Addition of anhydrotetracycline (ahTET) to the culture media leads to a DSB at the I-SceIcs. Indeed, two hours after ahTET addition, quantitative PCR amplification of genomic DNA using primers flanking the I-SceIcs decreased to 40–50% of control levels in wild-type, *taz1*Δ, *rap1*Δ, and *poz1*Δ backgrounds, demonstrating that DSBs were induced in these strains with similar efficiencies (Fig 6B). With this system, we examined how efficiently the wild-type and mutant cells could repair the DSB. We transiently induced DSB formation at I-SceIcs by culturing cells in liquid media containing ahTET for two hours. After that, ahTET was washed out and the cells were spread onto ahTET-free plate media. Cells that were unsuccessful in repairing the I-SceI DSB (or healing it, e.g. by *de novo* telomere addition) would not form colonies. We examined genomic DNA extracted from 10 colonies each from wild type, *taz1*Δ, *rap1*Δ strains and confirmed that none of them contained mutation in I-SceIcs, indicating that GCRs were not involved in generating survivors. Subsequent to the transient DSB induction in wild-type cells, the frequency of colony formation decreased to 62% of control (uncut) levels (Fig 6C). Strikingly, *taz1*Δ and *rap*1Δ cells showed further lower viabilities (37% and 33%, respectively). This result suggests that Taz1 and Rap1 promote DSB repair. *taz1*Δ *pof8*Δ cells showed similar survival with *taz1*Δ, consistent with the idea that the survivors occur not through telomerase-mediated GCRs, but through DSB repair, and suggesting that Taz1 promotes DSB repair independently of telomerase regulation. Interestingly, the BRCT domain-lacking *rap1*-A mutant showed significantly lower survival (42%) than wild-type, indicating that the Rap1 BRCT domain plays a significant role in DSB repair. We note, however, that the survival rate of the *rap1-*A strain was still slightly but significantly higher than *rap*1Δ, suggesting that Rap1 is involved in two pathways that promote DSB repair: one BRCT-domain-dependent and the other independent (Fig 6D). We examined whether Taz1 and Rap1 physically bind to the DSBs by chromatin immunoprecipitation (ChIP). No significant ChIP signal was detected for both Taz1 and Rap1 at the sites 1.5 and 5 kb apart from I-SceIcs at 2 and 4 hrs after DSB induction, while they localized at telomeres (S6B Fig). It is possible that Taz1 and Rap1 were only transiently recruited to DSBs, which made the ChIP detection difficult. Alternatively, Taz1 and Rap1 are indirectly involved in the DSB repair (see Discussion).

To examine if the impaired DSB repair in *taz1*Δ and *rap*1Δ cells leads to GCRs, we measured GCR frequencies caused by the I-SceI-induced DSB. We allowed the I-SceI endonuclease to be continuously active by culturing cells on plate media containing ahTET. Under this condition, faithful DSB repair would be detrimental to cell viability because it would regenerate I-SceIcs, leading to incessant cut and repair cycles. In contrast, I-SceIcs would become resistant to I-SceI cleavage when the I-SceIcs was lost via mutagenic DSB repair, including GCRs, indels, and point mutations. In the wild-type background, only 0.58% of cells survived and formed colonies, suggesting that GCRs and erroneous DNA repair is rare (Fig 6F). The survival was likely caused by *de novo* telomere addition, because *trt1*Δ showed significantly lower survival rates than wild type (Fig 6E). *ccq1*Δ also decreased the survival rate to a similar level shown by *trt1*Δ, suggesting that Ccq1 is required for *de novo* telomere addition at DSBs, as in the case of telomerase-mediated telomere elongation at native telomeres. In contrast, a higher fraction of cells survived in the *taz1*Δ and *rap1*Δ backgrounds. In all wild-type, *taz1*Δ, and *rap1*Δ survivors, in which the breakpoints were identified (n = 9, 9, and 7, respectively), the I-SceIcs was eliminated by terminal deletion associated with *de novo* telomere addition, suggesting that the observed viabilities reflect frequencies of terminal deletions in wild-type, *taz1*Δ and *rap1*Δ strains. Therefore, Taz1 and Rap1 not only promotes faithful DSB repair (Fig 6C), but also prevents erroneous DSB repair by suppressing terminal deletion coupled with *de novo* telomere addition. In wild-type cells, breakpoints of the deletions were close to (1~10 bp) I-SceIcs in all 9 clones in which the breakpoints were identified (Fig 6G). In contrast, breakpoints of 4 out of 9 clones in the *taz1*Δ survivors and 5 out of 7 clones in *rap1*Δ survivors were within this range, but the breakpoints in the other 5 *taz1*Δ survivor clones (one was ~300-bp centromeric, and four were 9~13-kb centromeric to the I-SceIcs) and two *rap1*Δ clones were located far (>10 bp) from the I-SceIcs. Therefore, we monitored DNA resection around DSBs indirectly through ChIP experiments of RPA. Localization of RPA subunit Rad11 was increased 2 hours after ahTET addition at 1.5 kb distant from I-SceIcs, while it was increased 4 hours at 5 and 13 kb both in wild type and *taz1*Δ strains (S6C Fig). These results suggest that Taz1 and Rap1 repress *de novo* telomere addition associated with DNA resection. The uncontrolled telomere addition may have contributed to the higher frequency of chromosome deletions found in *taz1*Δ and *rap1*Δ compared to wild-type clones (Fig 6E and 6F). As *rap1-A* and *poz1*Δ strains did not show increased survival, disruption of either Rap1 BRCT domain or Rap1-Poz1 pathway is not sufficient for inducing mutagenic DNA repair, and they function redundantly for suppressing telomere addition. We also examined distribution of breakpoints of telomere addition in *rap1-A* survivors. Since the breakpoints often located far from I-SceIcs (>10 bp, 3/6) similarly with *taz1*Δ and *rap1*Δ, the broader distribution of breakpoints shown in the shelterin mutants would be caused consistently by a loss of the Rap1 BRCT domain-dependent DSB repair pathway. Collectively, these results suggest that Taz1 represses GCRs by facilitating proper DSB repair and suppressing *de novo* telomere addition.

In a previous research, Taz1 was implicated in DSB repair because *taz1*Δ cells were sensitive to DNA damaging agents, such as methyl methane sulfonate (MMS) and bleomycin [31]. The sensitivity was augmented by simultaneous deletion of *cds1*^+^ [31]. We tested if a loss of the impaired DSB repair was responsible for the higher GCR rates in *taz1*Δ and *rap1*Δ. We found that the increased GCR rates and sensitivity to DNA-damaging reagents were not always correlated: *rap1*Δ was not sensitive to bleomycin (S6D Fig), consistent with a study showing that *rap*1Δ is not sensitive to MMS [36]. Moreover, *taz1*Δ *cds1*Δ and *rap1*Δ *cds1*Δ double-mutant strains showed decreased GCR rates relative to *taz1*Δ and *rap1*Δ (S6E Fig). These results indicate that the high GCR rates in *taz1*Δ and *rap1*Δ is not simply due to defective DSB repair.

## Discussion

Here we have described the hitherto unappreciated functions of the shelterin components Taz1 and Rap1 in the maintenance of genome integrity. Deletion of Taz1 and Rap1 led to increased spontaneous formation of GCRs, especially those involving chromosome terminus deletions associated with *de novo* telomere addition. Deregulation of telomerase accessibility appeared to be essential for frequent GCRs in *taz1*Δ and *rap1*Δ strains. Importantly, disruption of either the Rap1-Poz1 association (ΔP in Fig 5) or the BRCT domain of Rap1 did not induce high GCR rates (mutant A in Fig 5). By contrast, simultaneous deletion of the Rap1 BRCT domain and Poz1-binding domain led to higher GCR rates (Fig 5B). These results indicate that Rap1 suppresses GCRs through two independent pathways; one is BRCT domain-dependent, and the other is via a Rap1-Poz1 interaction that contributes to suppressing telomerase recruitment. These two pathways compensate for each other to suppress GCRs because inactivation of either one did not significantly affected the GCR rate, but simultaneous inactivation of both increased GCR rates in our system (Fig 5B).

### How telomeric proteins Taz1 and Rap1 suppress GCRs at non-telomeric regions

How do shelterin components Taz1 and Rap1 repress GCRs at the breakpoint region? Although it has been reported that Taz1 can be recruited to telomere-like sequences outside telomeres, the breakpoint region of our GCR assay system does not have any telomeric DNA motif and a previous genome-wide chromatin immunoprecipitation analysis failed to detect Taz1 in the breakpoint region [49]. In addition, other genome-wide studies have indicated that telomeres are unlikely to reside stably in close proximity to the breakpoint region by examining 3D chromosome positioning in the nucleus [50, 51]. One possibility is that the higher GCR rates observed in the *taz1*Δ and *rap1*Δ strains were caused indirectly from the abnormal telomeres in these mutants. For example, aberrantly elongated telomeres (*taz1*Δ and *rap1*Δ) or gapped telomeres (*taz1*Δ) sequester substantial amounts of DSB repair factors [52], thereby compromising DSB repair efficiency outside telomeres. However, we showed that Taz1 still significantly suppressed GCRs in cells with no telomeric DNA (circular chromosomes) (Fig 4B). This result favors another hypothesis that DSBs directly recruit Taz1 and Rap1 in a telomeric DNA sequence-independent manner, rather than the indirect model. However, we have not detected any localization of Taz1 and Rap1 at DSBs in ChIP experiments (S6B Fig). It is possible that although Taz1-Rap1 associates with DSBs, the association is very limited either temporally or stoichiometrically, which made detection by the ChIP experiment difficult. In humans, it was reported that human TRF1 and TRF2 are recruited to DNA damage sites to promote homologous recombination-directed DSB repair (HDR). It is known that the association happens only transiently immediately after DSB induction [53–55]. In budding yeast, inner nuclear envelope protein Mps3 binds unrepaired DSBs, thereby spatially recruiting them close to the nuclear envelope [56].

### Taz1 and Rap1 promote non-telomeric DSB repair

We showed that Taz1 and Rap1 promote survival after a transient site-specific DSB induction, suggesting that they are involved in DSB repair. Notably, the experiment with constitutive DSB induction showed that GCR breakpoints in *taz1*Δ were in some cases far (> ~10 kb) from the original break site. These large deletions in *taz1*Δ can be explained by excessive resection of DSB ends or defective HDR. Firstly, Taz1 might suppress DNA end resection at non-telomeric DSB sites. This is not surprising given that Taz1 and Rap1 suppress extensive resection at telomeres [57]. Excessively resected DNA may provoke loss of the opposite strand, because it is known that 3’-end strands are degraded several hours after resection of the 5’ strand at a DSB in budding yeast [58]. It is possible that *de novo* telomere addition occurred at such new DSB sites distant from the original DSB site, which may account for the *de novo* telomere additions that are far from the original DSB site in *taz1*Δ cells. Alternatively, since the Taz1 homolog TRF1 promotes HDR at non-telomeric DSBs [52, 53], it is possible that Taz1 suppresses large deletions by promoting HDR. According to this scenario, in the *taz1*Δ strain, cells that engage in grossly defective HDR to repair DSBs are inviable, but an increased accessibility of telomerase to DSBs would promote formation of *de novo* telomere addition to the DSBs. In addition, the longer reaction time required by the inefficient HDR in *taz1*Δ, compared to *taz1*^+^, would also lead to extensive resection, resulting in large deletions. Similar to our results, budding yeast *pif1* mutants, in which *de novo* telomere addition is highly promoted, showed large deletions concomitant with spontaneous and HO endonuclease-induced *de novo* telomere addition [52]. This also can be explained by inefficient HDR, because it was recently shown that Pif1 promotes HDR [58].

### Functions of the Rap1 BRCT domain for DSB repair and GCR suppression

Previous studies with budding yeast and mammalian cells have proposed that telomere-binding proteins prevent GCRs solely by suppressing fatal inter-chromosomal fusions or *de novo* telomere additions. Our results, however, raise the possibility that components of the shelterin complex have a previously unappreciated mechanism for suppressing genome instability. Remarkably, our Rap1 truncation analysis demonstrated that the previously uncharacterized BRCT domain together with the Poz1-binding domain suppresses GCRs. Although we do not know at this moment the detailed molecular mechanism of GCR suppression by the BRCT domain, it does not depend on NHEJ because DNA ligase IV is dispensable for the increased GCR rate in *rap1*Δ. The BRCT domain-dependent mechanism would also be independent of telomerase regulation, given that the Rap1-A strain lacking the BRCT domain has normal telomere lengths [27]. Rather, the BRCT domain would be involved in DSB repair, because the Rap1-A strain showed reduced survival after transient DSB induction. Although the N-terminal BRCT domain is a conserved feature of Rap1 among other species, including budding yeast and humans, its function has been unclear, except that a mutation in the BRCT domain of budding yeast Rap1 affects its regulatory activity related to transcription [59]. It would be interesting to examine whether the BRCT domain of mammalian Rap1 is involved in suppression of genome instability or promotion of DSB repair. Further analysis of fission yeast shelterin components and their counterparts in mammals will reveal the detailed mechanism of the GCR suppression.

## Materials and methods

### *S*. *pombe* strains, plasmids, and antibodies

All of the experiments were performed using the *S*. *pombe* strains listed in S1 Table. Growth media, basic genetics, and construction of strains carrying deletion alleles or epitope-tagged proteins were described previously [60]. Point mutations were introduced using a QuikChange Multi Site-Directed Mutagenesis Kit (Agilent, 200514), or by manually using *Dpn* I and PCR. All plasmids constructed in this study were sequence-verified.

*TK* was cloned from FY2317 [61] and was flanked by a cytomegalovirus promoter and *S*. *cerevisiae LEU2* terminator. *ura4*^+^ and *TK* were inserted between SPAC29B12.14c and SPAC1039.01 on chromosome I (at nt 5442736 of the chromosome I sequence described in Pombase: http://www.pombase.org/)

For immunoblot of Rap1 and Cdc2, polyclonal anti-Rap1 antibody [29] and Cdc2 p34 [PSTAIRE] antibody (sc-53, Santa Cruz Biotechnology, Inc.) were used.

### Measurement of GCR rates

For measurements of GCR rates, a previously described method using fluctuation analysis in budding yeast was applied to fission yeast, with some modifications [62]. Cells were streaked on YES agar plates and incubated at 32°C to form single colonies. Whole colonies were picked up by excising colonies with a sterile scalpel, suspended in YES liquid media, and incubated at 32°C until saturation. A portion of the saturated cells (at most 500 μl per plate) was plated on a YES agar plate supplemented with 1 mg/ml 5-FOA and 20 μg/ml FUdR. At the same time, 100 μl of a 10^5^-fold dilution of the cell suspension was plated on another YES agar plate. Both plates were placed at 32°C, and colony numbers of selective and non-selective media were counted after 5- and 2-day incubations, respectively. The total number of GCRs in each liquid culture was estimated from the colony numbers using a following equation [63]:

m=(r/z‐0.693)/ln(r/z+0.367)

where m represents a number of GCRs formed de novo, not formed by duplication of pre-formed GCRs, in each total liquid culture, r represents the number of colonies on the selective media, and z represents the fraction of cells plated on the selective media.

This procedure was performed with at least 7 independent colonies, and median values of [(number of GCRs: m) / (total cell number)] were shown as the GCR rate. When the median value was zero, a tentative median value was calculated by assuming m = 1 in all colonies and shown in figures as an upper bound, as described previously [62]. Confidence intervals for the GCR rates were calculated as previously described [62], and shown in graphs as error bars. In order to determine statistical significance of differences in GCR rates, a Mann-Whitney test was performed.

### Determination of locations and sequences of GCR breakpoints

To determine breakpoint sites in GCR survivors, breakpoint sequences were mapped to ~400 bp resolution by sequential PCR analysis, as previously described [25] (S7A Fig). For survivors with no loss of amplification by any primer sets, PCR products spanning *ura4*^+^ and *TK* (in GCR assay) or I-SceIcs (in the site-specific DSB assay) were sequenced to check whether they contained point mutations. To assess whether *de novo* telomere addition had occurred, PCR analysis was performed with a primer designed to anneal to a sequence centromeric from breakpoint and a primer including telomere sequence (M13R-T1: 5’-caggaaacagctatgacctgtaaccgtgtaaccgtaac-3’ or M13R-19: 5’-caggaaacagctatgaccctgtaaccccctgtaacc-3’; the underlined sequence was added to increase specificity of amplification after the 2nd cycle) using iProof DNA polymerase (Bio-Rad) (S7B Fig) [64]. Reaction mixtures were incubated at 98°C for 30 s, then cycled 30 times at 98°C for 10 s, 68°C for 20 s, and 72°C for 1 min. For survivors without *de novo* telomere addition, the breakpoint sequence was determined by ligation-mediated PCR, as described previously [65] (S7C Fig).

### Measurements of GCR frequencies after G1 arrest

To examine how G1 arrest affects GCR frequency, we modified the protocol for measurement of GCR frequencies after treatment with DNA damaging agents [66]. Cells exponentially grown in YES liquid medium were divided equally, washed twice with EMM medium with/without ammonium chloride (nitrogen source), and incubated with the same medium for 24 hours. The incubated cells were washed once with YES medium and incubated with YES liquid medium overnight until saturation. Defined numbers of cells were plated on YES plates containing/lacking 5-FOA and FUdR. Resulting colony numbers were counted, and the ratio of surviving cells in 5-FOA and FUdR was calculated to obtain the respective GCR frequencies.

### Site-specific double-strand break assay

Site-specific DSB induction was performed essentially as described previously [67], with the following modifications. The tetracycline-inducible I-SceI integration plasmid containing *LEU2* was integrated at the *leu1-32* locus. A plasmid containing the I-SceI cleavage site as well as *ura4*^+^ and hygromycin B selectable markers was integrated at the same site as the *ura4*^+^-*TK* cassette in our GCR assay strain. DSB was induced by addition of ahTET (Sigma, 3 μM final).

### Quantitative PCR (qPCR)

qPCR was performed using a StepOnePlus real-time PCR system (Applied Biosystems). Sequences of PCR primer sets are listed in S2 Table.

### Chromatin immunoprecipitation (ChIP)

ChIP assays were performed essentially as described previously [13], with the following modifications. The cell concentration was adjusted to 1.0 × 10^7^ cells/mL just before addition of ahTET and a defined volume of the culture was collected at indicated time points for fixation. Immunoprecipitation was performed with anti-myc antibody (9B11, Cell Signaling) using Dynabeads M‐280 Sheep anti‐Mouse IgG (Invitrogen). DNA was purified and extracted from washed beads and input samples with Chelex 100 Resin (BioRad) as described previously [68], and analyzed by qPCR.

### Estimate of double point mutation rate of *ura4*^+^ and *TK*

According to a previous report [26], fission yeast acquires 5-FOA resistance by spontaneous inactivating point mutation of either *ura4*^+^ or *ura5*^+^, and the rate of spontaneous mutation which confers 5-FOA resistance is 1.3 × 10^−7^ (/cell division). Among these mutations, ratio of mutation in ura5/ura4 is 1.85, so inactivating mutation rate of *ura4*^+^ can be calculated as 1.3 × 10^−7^ / (1.85+1) = 4.6 × 10^−8^. Given that the length of ORF of *ura4*^+^ and *TK* are ~800 and ~1100 bp, respectively, we estimated mutation rate of *TK* as 4.6 × 10^−8^ × (1100/800) = 6.3 × 10^−8^. If this estimate is true, the rates of point mutations that confer 5-FOA and FUDR resistance are both nearly or less than 10^−7^ per cell division.

## Supporting information

S1 Table Fission yeast strains used in this study. (DOCX)

S2 Table Sequences of primers used in this study. (DOCX)

S3 Table List of GCR rates and confidence intervals. (DOCX)

S4 Table Sequences of breakpoint junctions in GCR clones. (DOCX)

S5 Table List of original data used to calculate GCR rates. (XLSX)

S1 Fig Related to Fig 1. (A) Sensitivity of cells with *ura4*^+^ and/or *TK* to 5-FOA and/or FUdR. The wild-type strain in this experiment has neither *ura4*^+^ nor *TK*. Cells diluted by 1:10 serial dilutions were spotted on YES agar plates containing the indicated drugs and incubated at 32°C. (B) Locations of GCR junctions in the breakpoint region. Subscripts at arrowheads correspond to the numbers assigned to each GCR survivor in S2 Table. Nucleotide coordinates of the 3’ end of *sec16*^+^ and the 5’ end of inserted *ura4*^+^ are shown. Closed and open arrowheads indicate deletion and translocation types, respectively. (C) Schematic representation of the translocation observed in GCR survivors derived from wild-type cells. In these strains, the breakpoint region was joined to the chromosome 1 left arm in the opposite orientation. We have not addressed whether the original chromosome 1 left arm was retained or not. (D) GCR rates of strains expressing wild type or mutant Pfh1. Pfh1 constructs were expressed from original *pfh1*^+^ promoter integrated on genome. Asterisk and NS represent P<0.05 and P>0.05, respectively, relative to wild type.
(TIF)

S2 Fig Related to Fig 2. GCR rates of wild-type, *taz1*Δ, and *rap1*Δ strains incubated in 32°C and 20°C.
(TIF)

S3 Fig Related to Fig 3. (A) GCR frequency of indicated strains in the presence and absence of nitrogen source in media. Please note that the GCR frequencies shown in this figure was measured by a different method from GCR rates shown in the other figures (see Methods). (B)(C) GCR rates of strains lacking essential factors for heterochromatin (B), and a *poz1* point mutant that is defective in heterochromatin at telomeres (C).
(TIF)

S4 Fig Related to Fig 4. Maintenance of circularization of chromosome I & II in *trt1*Δ strains after Trt1 re-expression. *Not*I-digested chromosomes were analyzed by pulsed-field gel electrophoresis and Southern blotting. The terminal fragments of chromosome I and II were detected using a mixture of four probes detecting C, I, L, & M chromosome fragments.
(TIF)

S5 Fig Related to Fig 5. Expression of the Rap1 proteins. The whole cell extracts were analyzed by immunoblotting using anti-Rap1 antibody and anti-PSTAIR antibody for Cdc2 (loading control). WT, wild-type; and Δ, *rap1*Δ. Asterisks indicate non-specific bands.
(TIF)

S6 Fig Related to Fig 6. (A) Efficiency of DSB induction in I-SceIcs in an experiment conducted with the same protocol of Fig 6B, except that cells were washed out ahTET two hours after ahTET addition. N = 1. (B)(C) Localization of Taz1, Rap1, and Rad11 around I-SceI cut site. Cells expressing (B) Taz1-myc or Rap1-myc and (C) Rad11-myc were examined by ChIP using anti-myc antibody. Mean values ± SEM are indicated. N = 3. (D) Sensitivity of wild type, *taz1*Δ, and *rap1*Δ strains to bleomycin. Cells serially diluted by 1:5 were spotted on YES agar plates with indicated drug and incubated at 32°C. (E) GCR rates of wild-type, *taz1*Δ, and *rap1*Δ strains in the presence or absence of *cds1*^+^.
(TIF)

S7 Fig Characterization of GCR breakpoints. Schematic representation of the strategy for characterizing GCR breakpoints. (A) Sequential PCR to narrow down the location of breakpoints. Solid and empty thick lines represent original and newly added sequences, respectively. (B) Stretch PCR to assess telomere addition [61]. (C) Ligation-mediated PCR to assess translocation. See Materials and Methods for details.
(TIF)

S8 Fig I-SceIcs-containing cassette coordinate used in S4 Table. (TIF)
