# Supplementary material for: Telomere-binding proteins Taz1 and Rap1 regulate DSB repair and suppress gross chromosomal rearrangements in fission yeast
Source: PLoS Genet. 2019 Aug 27;15(8):e1008335. doi: 10.1371/journal.pgen.1008335 (PMC6733473; doi:10.1371/journal.pgen.1008335)
Supplement: S1 Table — (DOCX) [file pgen.1008335.s001.docx]

**S1 Table. Fission yeast strains used in this study.**

All strains except for JK317, JK320, and FY2317 were constructed for this study.

Strain Genotype

Fig 1B

HI4083 *h^-^ leu1-32 ura4-D18 Ch.1-5442737*::*CMV-TK/ura4^+^*

HI4085 HI4083 *rad2*::*kanMX6*

HI4087 HI4083 *mre11*::*kanMX6*

Fig 1C, D, & E

HI4083 *h^-^ leu1-32 ura4-D18 Ch.1-5442737*::*CMV-TK/ura4^+^*

Fig 2B

HI4083 *h^-^ leu1-32 ura4-D18 Ch.1-5442737*::*CMV-TK/ura4^+^*

HI4123 HI4083 *taz1*::*kanMX6*

HI4126 HI4083 *rap1*::*kanMX6*

HI4206 HI4083 *rap1*::*kanMX6 taz1*::*hphMX6*

HI4408 HI4083 *poz1*::*kanMX6*

HI4423 HI4083 *stn1-1*::*LEU2*

Fig 2C & D

HI4123 HI4083 *taz1*::*kanMX6*

HI4126 HI4083 *rap1*::*kanMX6*

Fig 2E

HI4083 *h^-^ leu1-32 ura4-D18 Ch.1-5442737*::*CMV-TK/ura4^+^*

HI4408 HI4083 *poz1*::*kanMX6*

HI4421 HI4083 *tpz1-I501A,R505E-3Flag*:*LEU2*

HI4123 HI4083 *taz1*::*kanMX6*

HI4339 HI4083 *taz1*::*kanMX6 poz1*::*hphMX6*

HI4804 HI4083 *taz1*::*kanMX6 tpz1-I501A,R505E-3Flag*:*LEU2*

HI4126 HI4083 *rap1*::*kanMX6*

HI4796 HI4083 *rap1*::*kanMX6 poz1*::*hphMX6*

HI4809 HI4083 *rap1*::*kanMX6 tpz1-I501A,R505E-3Flag*:*natMX6*

Fig 3A

HI4083 *h^-^ leu1-32 ura4-D18 Ch.1-5442737*::*CMV-TK/ura4^+^*

HI4171 HI4083 *lig4*::*hphMX6*

HI4123 HI4083 *taz1*::*kanMX6*

HI4172 HI4083 *taz1*::*kanMX6* *lig4*::*hphMX6*

HI4126 HI4083 *rap1*::*kanMX6*

HI4173 HI4083 *rap1*::*kanMX6 lig4*::*hphMX6*

Fig 3B

HI4083 *h^-^ leu1-32 ura4-D18 Ch.1-5442737*::*CMV-TK/ura4^+^*

HI4227 HI4083 *bqt4*::*hphMX6*

HI4841 HI4083 *bqt4*::*hphMX6 leu1^+^*::*GFP–bqt4-*∆*N*

HI4842 HI4083 *bqt4*::*hphMX6 leu1^+^*:: *rap1-GFP–bqt4-*∆*N*

HI4249 HI4083 *bqt3*::*kanMX6*

HI4721 HI4083 *rap1^213/378/422/456/513A^*:*LEU2*

HI4332 HI4083 *lem2*::*kanMX6*

HI4333 HI4083 *man1*::*kanMX6*

HI4576 HI4083 *pku70*::*hphMX6*

Fig 3C

HI4083 *h^-^ leu1-32 ura4-D18 Ch.1-5442737*::*CMV-TK/ura4^+^*

HI4227 HI4083 *bqt4*::*hphMX6*

HI4123 HI4083 *taz1*::*kanMX6*

HI4839 HI4083 *taz1*::*kanMX6 bqt4*::*hphMX6*

HI4126 HI4083 *rap1*::*kanMX6*

HI4840 HI4083 *rap1*::*kanMX6 bqt4*::*hphMX6*

Fig 4A

HI4083 *h^-^ leu1-32 ura4-D18 Ch.1-5442737*::*CMV-TK-ura4^+^*

HI4843 HI4083 *pof8*::*hphMX6*

HI4123 HI4083 *taz1*::*kanMX6*

HI4844 HI4083 *taz1*::*kanMX6 pof8*::*hphMX6*

HI4126 HI4083 *rap1*::*kanMX6*

HI4845 HI4083 *rap1*::*kanMX6 pof8*::*hphMX6*

HI4085 HI4083 *rad2*::*kanMX6*

HI4846 HI4083 *rad2*::*kanMX6 pof8*::*natMX6*

Fig 4B & S4

HI4260 HI4083 *trt1*::*kanMX6*

HI4831 HI4083 *trt1*::*kanMX6 pREP1-trt1^+^*

HI4852 HI4083 *trt1*::*kanMX6 taz1*::*natMX6*

HI4853 HI4083 *trt1*::*kanMX6 taz1*::*natMX6 pREP1-trt1^+^*

HI4854 HI4083 *taz1*::*natMX6 trt1*::*kanMX6*

HI4855 HI4083 *taz1*::*natMX6 trt1*::*kanMX6 pREP1-trt1^+^*

HI4856 HI4083 *trt1*::*kanMX6 taz1*::*natMX6 poz1*::*hphMX6*

HI4857 HI4083 *trt1*::*kanMX6 taz1*::*natMX6 poz1*::*hphMX6 pREP1-trt1^+^*

HI4858 HI4083 *rap1*::*natMX6 trt1*::*kanMX6*

HI4859 HI4083 *rap1*::*natMX6 trt1*::*kanMX6 pREP1-trt1^+^*

Fig 5B

HI4083 *h^-^ leu1-32 ura4-D18 Ch.1-5442737*::*CMV-TK-ura4^+^*

HI4817 HI4083 *rap1-WT*:*LEU2*

HI4818 HI4083 *rap1-∆2-110*:*LEU2*

HI4820　　 HI4083 *rap1-∆2-174*:*LEU2*

HI4821 HI4083 *rap1-∆2-248*:*LEU2*

HI4822 HI4083 *rap1-∆2-300*:*LEU2*

HI4823 HI4083 *rap1-∆2-370*:*LEU2*

HI4824 HI4083 *rap1-∆2-456*:*LEU2*

HI4825 HI4083 *rap1-∆2-512*:*LEU2*

HI4849 HI4083 *rap1-∆457-512*:*LEU2*

HI4826 HI4083 *rap1-∆2-110, 457-512*:*LEU2*

HI4860 HI4083 *rap1-∆2-110, 457-512*:*LEU2* *pof8*::*hphMX6*

HI4126 HI4083 *rap1*::*kanMX6*

Fig 5C

HI4860 HI4083 *rap1-∆2-110, 457-512*:*LEU2* *pof8*::*hphMX6*

Fig 6B & S6C Fig

HI4706 *h^-^ ura4-D18 leu1-32*::*adh-TetR/CaMV35Sp-I-SceI/LEU2*

*Ch.1-5442737*::*I-SceIcs/hphMX6/ura4^+^*

HI4719 HI4706 *taz1*::*kanMX6*

HI4850 HI4706 *rap1*::*kanMX6*

HI4861 HI4706 *poz1*::*kanMX6*

Fig 6C

HI4706 *h^-^ ura4-D18 leu1-32*::*adh-TetR/CaMV35Sp-I-SceI/LEU2*

*Ch.1-5442737*::*I-SceIcs/hphMX6/ura4^+^*

HI4719 HI4706 *taz1*::*kanMX6*

HI4862 HI4706 *taz1*::*kanMX6 pof8*::*natMX6*

HI4850 HI4706 *rap1*::*kanMX6*

HI4851 HI4706 *rap1-∆2-110*:*natMX6*

Fig 6E & 6F

HI4706 *h^-^ ura4-D18 leu1-32*::*adh-TetR/CaMV35Sp-I-SceI/LEU2*

*Ch.1-5442737*::*I-SceIcs/hphMX6/ura4^+^*

HI4719 HI4706 *taz1*::*kanMX6*

HI4850 HI4706 *rap1*::*kanMX6*

HI4805 HI4706 *poz1*::*kanMX6*

HI4863 HI4706 *trt1*::*kanMX6*

HI4864 HI4706 *ccq1*::*kanMX6*

S1A Fig

JK317 *h^-^ leu1-32 ura4-D18* (lab stock)

JK320 *h^-^ leu1-32* (lab stock)

FY2317 *h^+^ ade6-M210 hENT1-leu1 ura4-D18 his7-366*::*adh-TK-his7* (Hodson et al., 2003)

HI4083 *h^-^ leu1-32 ura4-D18 Ch.1-5442737*::*CMV-TK/ura4^+^*

S1B & S2 Fig

HI4083 *h^-^ leu1-32 ura4-D18 Ch.1-5442737*::*CMV-TK/ura4^+^*

HI4123 HI4083 *taz1*::*kanMX6*

HI4850 HI4083 *rap1*::*kanMX6*

S1D Fig

HI4083 *h^-^ leu1-32 ura4-D18 Ch.1-5442737*::*CMV-TK/ura4^+^*

HI4123 HI4083 *taz1*::*kanMX6*

HI4866 HI4083 *pfh1-WT:LEU2*

HI4867 HI4083 *pfh1-mt*:LEU2*

S3B Fig

HI4083 *h^-^ leu1-32 ura4-D18 Ch.1-5442737*::*CMV-TK/ura4^+^*

HI4577 HI4083 *clr4*::*hphMX6*

HI4561 HI4083 *swi6*::*hphMX6*

HI4123 HI4083 *taz1*::*kanMX6*

HI4865 HI4083 *rap1*::*kanMX6*

HI4578 HI4083 *taz1*::*kanMX6 clr4*::*hphMX6*

HI4579 HI4083 *taz1*::*kanMX6 swi6*::*hphMX6*

HI4868 HI4083 *rap1*::*kanMX6 clr4*::*hphMX6*

HI4869 HI4083 *rap1*::*kanMX6 swi6*::*hphMX6*

S3C Fig

HI4083 *h^-^ leu1-32 ura4-D18 Ch.1-5442737*::*CMV-TK/ura4^+^*

HI4870 HI4083 *poz1-W209A*

HI4123 HI4083 *taz1*::*kanMX6*

S5 Fig

HI4817 HI4083 *rap1-WT*:*LEU2*

HI4826 HI4083 *rap1-∆2-110, 457-512*:*LEU2*

HI4818 HI4083 *rap1-∆2-110*:*LEU2*

HI4126 HI4083 *rap1*::*kanMX6*

HI4825 HI4083 *rap1-∆2-512*:*LEU2*

S6A Fig

HI4706 *h^-^ ura4-D18 leu1-32*::*adh-TetR/CaMV35Sp-I-SceI/LEU2*

*Ch.1-5442737*::*I-SceIcs/hphMX6/ura4^+^*

HI4871 HI4706 *taz1-13myc*::*kanMX6*

HI4872 HI4706 *rap1-13myc*::*kanMX6*

S6B Fig

HI4706 *h^-^ ura4-D18 leu1-32*::*adh-TetR/CaMV35Sp-I-SceI/LEU2*

*Ch.1-5442737*::*I-SceIcs/hphMX6/ura4^+^*

HI4873 HI4706 *rad11-13myc*::*kanMX6*

HI4874 HI4706 *taz1*::*natMX6 rad11-13myc*::*kanMX6*

S6C Fig

HI4083 *h^-^ leu1-32 ura4-D18 Ch.1-5442737*::*CMV-TK/ura4^+^*

HI4875 HI4083 *cds1*::*hphMX6*

HI4123 HI4083 *taz1*::*kanMX6*

HI4876 HI4083 *taz1*::*kanMX6 cds1*::*hphMX6*

HI4126 HI4083 *rap1*::*kanMX6*

HI4877 HI4083 *rap1*::*kanMX6 cds1*::*hphMX6*
