# Supplementary material for: Telomere-binding proteins Taz1 and Rap1 regulate DSB repair and suppress gross chromosomal rearrangements in fission yeast
Source: PLoS Genet. 2019 Aug 27;15(8):e1008335. doi: 10.1371/journal.pgen.1008335 (PMC6733473; doi:10.1371/journal.pgen.1008335)
Supplement: S2 Table — (DOCX) [file pgen.1008335.s002.docx]

**S2 Table. Sequences of primers used in this study**

| PP1F | ggatgtcatgagaaatagttgatggaag |
| --- | --- |
| PP1R | taaaatcatcaagtcatgtgaataaac |
| PP2F | caatgaattgttgtgagagagactgatg |
| PP2R | ggcatgggataagaagagaaataggataag |
| PP3F | ctttcctcgggatgagcgatagcataag |
| PP3R | gcgcttccctgctcttatccatccaac |
| PP4F | agacactcgtctccagatacccatgtc |
| PP4R | agttgagattggaagtggccagagttg |
| PP5F | gacaaccgaaagttcgatttc |
| PP5R | gagacaaaacgttgacgatgtac |
| PP6F | catccactttgtccaatccacttacat |
| PP6R | gaagagtgcgcattgtgtgcatcagcc |
| PP7F | gtctgctgataactcttcgtcgtctacaac |
| PP7R | catgcgccattgaagggctggatgtag |
| PP8F | tacagcgtcggttcctcaacaaatttc |
| PP8R | tgtgttgggatacgagtattggatatg |
| PP9F | cagaatgttcctcaacagcttc |
| PP9R | gaatccactactatgataactattc |
| PP10F | gctcttaatcgttttgtctttttgctttg |
| PP10R | aatgctccggtccttttcac |
| PP11F | ccaccaggttatcttacctccagggtatg |
| PP11R | tttggcttaaccattttcacag |
| PP12F | cctgggtaattccaccgaggattgtag |
| PP12R | actggcagcaagtcgtttac |
| PP13F | gtattgcaaattacccaccagggagaag |
| PP13R | cacttggagaacttgggtgc |
| PP14F | tcgaccataaccaatcttccaatttgg |
| PP14R | aaaagttcgatggaaattgccatagac |
| PP15F | agcgttgcttttcaaccgaaattgagg |
| PP15R | ggcaaattgatagtaaaactaacacaatgc |
| PP16F | cccatgcaaagtatactatgacaacaagc |
| PP16R | ctcgtttcaggctagatttcccttatg |
| PP17F | ttataattcgcaaatgattaaatttag |
| PP17R | ttcattatccattgatgtgctg |
| PP18F | aagccattaacgcgtccaatgtataacttg |
| PP18R | tttcaatcccctgttgcaaacttgaag |
| PP19F | ttgaattagacggttccatgcttcaac |
| PP19R | ccagctagaatttttaatgatgtgaactcg |
| PP20F | aaataaggccctgcgttatacatagaagg |
| PP20R | cagttttacatgtacaagggaagtattcgg |
| PP21F | ctatctcttactttgcagaatttgggatcg |
| PP21R | ataagcattaaattgcaatctagac |
| PP22F | cctgaatcaaagttcccatgatttatagtc |
| PP22R | attttctattcccttgaagagccaagc |
| PP23F | aatatggtgttccagtccatttatggc |
| PP23R | gaaggaattcccgttaagaaagtcagc |
| PP24F | cagaagattctttgatatcgatacctcctg |
| PP24R | catctggaacccgttagacttgcttaagag |
| PP25F | ctagaagatagtaaattccatgggcacatg |
| PP25R | gggttctttgtggcccattc |
| PP26F | agacgggaaaccaaatggcgacattgg |
| PP26R | tttcgttagtaattactaaactttgccgc |
| PP27F | ttcagccagtgtagtgcccggcttagc |
| PP27R | catcttgcttatgatatgaagcatgtgatc |
| PP28F | ctggtgttatatacaaaaaccggttttgctg |
| PP28R | aaccctctcttcccttcctatc |
| PP29F | aagcttagctacaaatcccac |
| PP29R | ccaacaattccttggcaatggg |
| PP30F | ccatcccagtttaactatgcttcgtcg |
| PP30R | tcgctaccgcagtttacaatcacttc |
| PP31F | acgtggtctcttgcttttggctg |
| PP31R | aagcttgtgatattgacgaaac |
| M13R-T1 | caggaaacagctatgacctgtaaccgtgtaaccgtaac |
| M13R-C19 | caggaaacagctatgaccctgtaaccccctgtaacc |
| Adaptor univ. | gtaatacgactcactatagggcacgcgtggtcgacggcccgggctggt |
| BfaI | taaccagccc |
| HinPLI/MspI/TaqI | cgaccagccc |
| ADP1 | gtaatacgactcactatagggc |
| ADP2 | actatagggcacgcgtggt |
| ADPSEQ | gtcgacggcccgggctggt |
| ade6F | ctgtcgccactgttgctatc |
| ae6R | tctccatagcagccaaaagg |
| 1.5kbF | ccatcccagtttaactatgcttcgtcg |
| 1.5kbR | ccaacaattccttggcaatggg |
| 3kbF | ttcagccagtgtagtgcccggcttagc |
| 3kbR | tttcgttagtaattactaaactttgccgc |
| 5kbF | gacataaaccggacaacattagc |
| 5kbR | cgatgatggaaagtcaggag |
| 13kbF | ttaatcgttttgtctttttgctttg |
| 13kbR | ggatctcgtatacgaaagtaccggtcg |
| telF | ctatttctttattcaacttaccgcacttc |
| telR | attaattgggtaacggagtaacaatataga |
| I-sceIcsF | agagaagctggttggaaggc |
| I-sceIcsR | cacgtgctgatcagatccga |
