# Supplementary material for: Telomere-binding proteins Taz1 and Rap1 regulate DSB repair and suppress gross chromosomal rearrangements in fission yeast
Source: PLoS Genet. 2019 Aug 27;15(8):e1008335. doi: 10.1371/journal.pgen.1008335 (PMC6733473; doi:10.1371/journal.pgen.1008335)
Supplement: S3 Table — (DOCX) [file pgen.1008335.s003.docx]

**S3 Table. Full list of GCR rates measured in this study**

| First appearance | Genotype | Median rate [95% CI] | N |
| --- | --- | --- | --- |
|  |  |  |  |
| Fig 1B | wild type | 2.6 [1.8-6.0]*10^(-9) | 12 |
|  | *rad*2Δ | 2.0 [0.6-7.6]*10^(-7) | 7 |
|  | *mre11*Δ | 3.8 [2.3-7.2]*10^(-7) | 7 |
| Fig 2B | *taz1*Δ | 1.1 [0.5-2.1]*10^(-7) | 14 |
|  | *rap1*Δ | 8.5 [4.8-15.1]*10^(-8) | 14 |
|  | *rap1-I655R* | 2.6 [0.2-4.1]*10^(-7) | 7 |
|  | *taz1*Δ *rap1*Δ | 1.2 [0.8-2.1]*10^(-7) | 14 |
|  | *poz1*Δ | 3.9 [0-12.6]*10^(-9) | 7 |
|  | *stn1-1* (25°C) | 1.6 [0.7-10.4]*10^(-8) | 17 |
| Fig 2E | *tpz1-I501A/R505E* | <8.7 [0-10.6]*10^(-9) | 7 |
|  | *taz1*Δ *poz1*Δ | 4.1 [2.4-6.6]*10^(-8) | 16 |
|  | *taz1*Δ *tpz1-I501A/R505E* | 1.4 [0-3.1]*10^(-8) | 7 |
|  | *rap1*Δ *poz1*Δ | <5.6 [0-10.3]*10^(-9) | 7 |
|  | *rap1*Δ *tpz1-I501A/R505E* | <5.1 [0-17.2]*10^(-9) | 7 |
| Fig 3A | *lig4*Δ | <7.6 [0-10.6]*10^(-9) | 7 |
|  | *taz1*Δ *lig4*Δ | 1.3 [1.0-2.7]*10^(-7) | 7 |
|  | *rap1*Δ *lig4*Δ | 1.5 [0.4-13.4]*10^(-7) | 7 |
| Fig 3B | *bqt4*Δ | 4.3 [1.7-7.1]*10^(-8) | 21 |
|  | *bqt4*Δ + Bqt4-ΔN | 2.1 [0.9-3.5]*10^(-8) | 10 |
|  | *bqt4*Δ + Rap1-Bqt4ΔN | 8.0 [0-12.3]*10^(-9) | 11 |
|  | *bqt3*Δ | 3.0 [2.9-5.8]*10^(-8) | 7 |
|  | *rap1-5E* | <4.1 [0-5.6]*10^(-9) | 7 |
|  | *lem2*Δ | 7.5 [0-43.0]*10^(-8) | 20 |
|  | *man1*Δ | <1.3 [0-12.9]*10^(-8) | 9 |
|  | *pku70*Δ | 2.9 [1.9-5.8]*10^(-8) | 9 |
| Fig 3C | *taz1*Δ *bqt4*Δ | 1.7 [1.4-3.3]*10^(-7) | 19 |
|  | *rap1*Δ *bqt4*Δ | 2.2 [0.8-3.4]*10^(-7) | 14 |
| Fig 4A | *pof8*Δ | <8.8 [0-10.9]*10^(-9) | 7 |
|  | *taz1*Δ *pof8*Δ | 6.5 [0-32.2]*10^(-9) | 7 |
|  | *rap1*Δ *pof8*Δ | <7.4 [0-9.8]*10^(-9) | 7 |
|  | *rad2*Δ *pof8*Δ | 6.8 [3.5-22.0]*10^(-8) | 14 |
| Fig 4B | *trt1*Δ | <6.2 [0-7.0]*10^(-9) | 7 |
|  | *trt1*Δ + Trt1 plasmid | <2.9 [0-63.0]*10^(-9) | 7 |
|  | *trt1*Δ *taz1*Δ | <4.1 [2.7-5.4]*10^(-8) | 7 |
|  | *trt1*Δ *taz1*Δ + Trt1 plasmid | 1.8 [0-5.8]*10^(-8) | 9 |
|  | *taz1*Δ *trt1*Δ | 1.7 [0-3.6]*10^(-8) | 7 |
|  | *taz1*Δ *trt1*Δ + Trt1 plasmid | 5.3 [3.0-9.8]*10^(-8) | 7 |
|  | *trt1*Δ *taz1*Δ *poz1*Δ | <1.8 [0-4.8]*10^(-8) | 7 |
|  | *trt1*Δ *taz1*Δ *poz1*Δ + Trt1 plasmid | <1.5 [0-3.1]*10^(-8) | 7 |
|  | *rap1*Δ *trt1*Δ | <2.0 [0-2.8]*10^(-8) | 7 |
|  | *rap1*Δ *trt1*Δ + Trt1 plasmid | <2.5 [0-3.7]*10^(-8) | 7 |
| Fig 5B | *rap1 (full length)* | <6.0 [0-8.0]*10^(-9) | 7 |
|  | *rap1-A* | <5.7 [0-12.9]*10^(-9) | 7 |
|  | *rap1-B* | <6.5 [0-7.3]*10^(-9) | 7 |
|  | *rap1-C* | <6.9 [0-10.1]*10^(-9) | 7 |
|  | *rap1-D* | 6.0 [0-26.5]*10^(-9) | 7 |
|  | *rap1-E* | 5.6 [0-17.6]*10^(-9) | 7 |
|  | *rap1-F* | <6.2 [0-11.6]*10^(-9) | 7 |
|  | *rap1-G* | 3.8 [0-14.7]*10^(-8) | 7 |
|  | *rap1*Δ*P* | <8.7 [0-19.4]*10^(-9) | 7 |
|  | *rap1-A*Δ*P* | 9.5 [4.1-14.1]*10^(-8) | 7 |
|  | *rap1-A*Δ*P pof8*Δ | <7.9 [0-15.2]*10^(-9) | 7 |
| S1D Fig | *pfh1-WT* | <1.8 [0-2.9]*10^(-8) | 7 |
|  | *pfh1-mt** | 7.4 [2.8-19.8]*10^(-8) | 7 |
| S2 Fig | wild type (20°C) | 6.8 [0-16.6]*10^(-9) | 7 |
|  | *taz1*Δ (20°C) | 2.8 [0.5-5.5]*10^(-6) | 7 |
|  | *rap1*Δ (20°C) | 4.2 [1.2-6.2]*10^(-8) | 7 |
| S3B Fig | *swi6*Δ | 2.1 [1.3-3.4]*10^(-8) | 9 |
|  | *clr4*Δ | 8.9 [0-17.4]*10^(-9) | 14 |
|  | *taz1*Δ *swi6*Δ | 1.8 [1.1-2.6]*10^(-7) | 7 |
|  | *taz1*Δ *clr4*Δ | 6.1 [4.0-14.3]*10^(-8) | 9 |
|  | *rap1*Δ *swi6*Δ | 9.2 [3.3-15.3]*10^(-8) | 7 |
|  | *rap1*Δ *clr4*Δ | 1.8 [0-13.1]*10^(-8) | 7 |
| S3C Fig | *poz1-W209A* | 3.9 [1.8-7.3]*10^(-9) | 7 |
| S6E Fig | *cds1*Δ | <9.2 [0-11.3]*10^(-9) | 7 |
|  | *taz1*Δ *cds1*Δ | <1.0 [0-1.4]*10^(-8) | 7 |
|  | *rap1*Δ *cds1*Δ | 7.8 [0-102.3]*10^(-9) | 7 |
