# Supplementary material for: Telomere-binding proteins Taz1 and Rap1 regulate DSB repair and suppress gross chromosomal rearrangements in fission yeast
Source: PLoS Genet. 2019 Aug 27;15(8):e1008335. doi: 10.1371/journal.pgen.1008335 (PMC6733473; doi:10.1371/journal.pgen.1008335)
Supplement: S4 Table — (DOCX) [file pgen.1008335.s004.docx]

**S4 Table. Sequences of breakpoint junctions in GCR clones.**

Wild type (Fig. 1)

Telomere addition (12/15)

1. AGCTTCCTCCTCCCATTCACtacaggggttacaggttaca [5430820]

2. TTCGTTAATAGATTTCCTCAggttacggttacacggttac [5430888]

3. TCGAATGTAAGGGGAAGAATtacggttacggttacggtta [5433266]

4. CCAGCGTCTAGTGAGACCGGttacggttacggttacggtt [5433584]

5. GAACTATTTAACGGTGTTAAcggatccggatacccggata [5433955]

6. TCAACCGAAATTGAGGTTATaggttacggttacggttaca [5434625]

7. CAAAAAATCTCCGTGTTACTggttacggttacggttacag [5435002]

8. GTGTTACTTAATAAGATTCCggttacggttacggttacgg [5435014]

9. GGGTTGCAGAGTATAGTATGgttacggttacggttacagg [5437382]

10. GGGTTGCAGAGTATAGTATGgttacggttacggttacacg [5437382]

11. GAAAAAGCCAACCCATTGGTtacaggggttacaggttaca [5441047]

12. GTGTTATATACAAAAACCGGttacggttacacggttaacg [5441484]

Translocation (2/15)

1. GAGTCATAGCTG**ACTTTCTT**AACGGGAATTCCTTCGGTTT [5439913-5439952]

TACCGTTTCATC**ACTTTCTT**TTTATTTTATTAGTTAAAGC [110327-110288]

(opposite)

2. ATACCGTCAAGCT**ACAATAT**GCATCTGGTGTGTACAAAAT [*ura4* 1279]

CTAGATTTTATGC**ACAATAT**CCATCTGATAAGTAAAATCG [86935-86896]

(opposite)

Unidentified (1/15)

*taz1*∆ (Fig. 2)

Telomere addition (11/11)

1. AAGCATCTCCCAGGGTAGTTacggttacaggggttacagg [5437298]

2. ATCCTTCAAGTTTGCAACAGttacggtacacggttacaga [5436976]

3. GATAAAATCAGCAAAAAGGGtaacagggggttacagggtt [5434643]

4. AGCGACATAATTCCATGGTCggttacacggttacaggtta [5441431]

5. ATATCTTATTGTTTGCATTAcggttacaggttacggttac [5435587]

6. AAAAATCTCCGTGTTACTTAcggttacaggttacggttac [5435004]

7. ACCTAGATAATGGGTTGCAGgggttacggttacggttaca [5437371]

8. ATCCTTCAAGTTTGCAACAGttacggttacacgggttaca [5436976]

9. TGGGACGTGGTCTCTTGCTTggttacaggggttacaggtt [*ura4* 444]

10. GTTATGTGGTATCCGAATCAggttacagggttacaggtta [5438926]

11. TCTTACCTCCAGGGTATGATtgtggttacggttacacggt [5431981]

*rap1*∆ (Fig. 2)

Telomere addition (10/10)

1. GACTAGAAATCTGTTGCGGTtacacggttacaggttacgg [5437795]

2. ATAATTTGCTCCAAAAAAGGttacggttacaggttacggt [5436940]

3. CCTCAACAAATTTCTAACGGttacaggttacagggttacg [5430102]

4. GTCTTAAGCATCTCCCAGGGtacggttacacggttacaca [5437293]

5. ACACATAAGAAAATTACTACggttacaggggttacggtta [5432001]

6. AAATTAGGTACGTTCGGAGTacggttacagggggttacgg [5427534]

7. TTTCTTTGTTTCTTTGTCTTataggttacaggtt [5428816]

8. AATGGGTTGCAGAGTATGGTtacagttacggttacagggt [5439379]

9. TGTGGTATCCGAATCATGAAcggttacaggggttacaggt [5438930]

10. AGATGAATTGGTCCGAAAAGttacggttacacggttacac [5434196]

rap1-A∆P (Fig. 5)

Telomere addition (9/10)

1. ATCTTACCTCCAGGGTATGAtacggttacaggggggttac [5431980]

2. CAGTAATTGACGCGGGGAGTtacggttacaggggttacgg [5436557]

3. GCTTGTTGCACTTTGCGGTTacaggttacggttacagggt [5431079]

4. AAAAGGTGGAAGAAGCGAACggttacaggggtacggttac [5427817]

5. CTAGAAATCTGTTGCGGTCTgggttacaggttacagggtt [5437797]

6. GTTACCTGTGCTAAGTGCCGgttacagggttacagggtta [5430641]

7. AAAAATCTCCGTGTTACTTAcggttacaggttacagttac [5435004]

8. TCCACCATGTTTGCTTCTTCggttacggttacggttacgg [5429865]

9. GGAAGTCAGCAATTTTTGTTggttacggttacggttacgg [5437275]

Unidentified (1/10)

Wild type (Fig. 6)

Telomere addition (9/10)

1. GCAGAGTTACGCTAGGGATAcggttacaggttacggttac [cc1351]*

2. CCTGCAGAGTTACGCTAGGGgttacggttacggttacacg [cc1348]*

3. TGCAGAGTTACGCTAGGGATacggttacaggttacggtta [cc1350]*

4. ACCTGCAGAGTTACGCTAGGttacggttacggttacggtt [cc1347]*

5. ACCTGCAGAGTTACGCTAGGttacggttacacggttacag [cc1347]*

6. CGTCGACCTGCAGAGTTACGgttacggttacaggttacgg [cc1342]*

7. GCAGAGTTACGCTAGGGATAcggttacggttacaggttac [cc1351]*

8. CGTCGACCTGCAGAGTTACGgttacaggggttacaggtta [cc1342]*

9. CGTCGACCTGCAGAGTTACGgttacaggttacggttacgg [cc1341]*

Unidentified (1/10)

*taz1*∆ (Fig. 6)

Telomere addition (9/10)

1. AATGTAAAATTTTTTTGGTTacagggttacacggttacac [cc924]*

2. GCAGAGTTACGCTAGGGATAcggttacaggttacggttac [cc1351]*

3. ACCTGCAGAGTTACGCTAGGttacggttacagggttacgg [cc1347]*

4. CCTCCAGGGTATGATACACAggttacaggttacggttacg [5431986]

5. GGTTACAAAAAATCTCCGTGttacaggttacggttacacg [5434997]

6. TCCCCACCAGGTTATCTTACggttacggttacaggggtta [5431967]

7. TCCCCACCAGGTTATCTTACggttacggttacagggttac [5431967]

8. GCAGAGTTACGCTAGGGATAcggttacagggttacggtta [cc1351]*

9. CCTGCAGAGTTACGCTAGGGttacggttacagggttacac [cc1348]*

Unidentified (1/10)

*rap1*∆ (Fig. 6)

Telomere addition (7/10)

1. ACCTGCAGAGTTACGCTAGGttacagggttacagggggtt [cc1345]*

2. GCAGAGTTACGCTAGGGATAcggttacaggggttacaggg [cc1351]*

3. GCAGAGTTACGCTAGGGATAcggttacaggggttacaggg [cc1351]*

4. GCAGAGTTACGCTAGGGATAcggttacaggggttacggtt [cc1351]*

5. GCAGAGTTACGCTAGGGATAcggttacaggttacacggtt [cc1351]*

6. ATTGAAAAAGTCGATGCCTTacggttacagggggttacag [cc948]*

7. TTACAAAAAATCTCCGTGTTcggttacaggggttacggtt [5434999]

Unidentified (3/10)

rap1-A (Fig. 6)

Telomere addition (6/9)

1. ATTAAGAATGGGCCACAAAGgttacagggttacagggtta [5441203]

2. GATATGGTAGAAAAACTGGTggttacaggttacagggggt [cc428]*

3. CCTGCAGAGTTACGCTAGGGttacaggggttacaggttac [cc1348]*

4. ACCTGCAGAGTTACGCTAGGttacacggttacaggttacg [cc1347]*

5. ACCTGCAGAGTTACGCTAGGttacacggttacaggttacg [cc1346]*

6. AAGTCAGCAATTTTTGTTGTtcggttacaggggttacagg [5437275]

Point mutation of I-SceIcs (2/9)

1.

original: TAGGGATAACAGGGTAAT

mutated: TAGG-ATAACAGGGTAAT

2.

original: TAGGGATAA-CAGGGTAAT

mutated: TAGGGATAAACAGGGTAAT

Unidentified (1/9)

*: “cc” represents cassette coordinate where breakpoint is located within a cassette with I-SceIcs. See S8 Fig. for detail.
