# Supplementary figures and images for: Telomere-binding proteins Taz1 and Rap1 regulate DSB repair and suppress gross chromosomal rearrangements in fission yeast
Source: PLoS Genet. 2019 Aug 27;15(8):e1008335. doi: 10.1371/journal.pgen.1008335 (PMC6733473; doi:10.1371/journal.pgen.1008335)

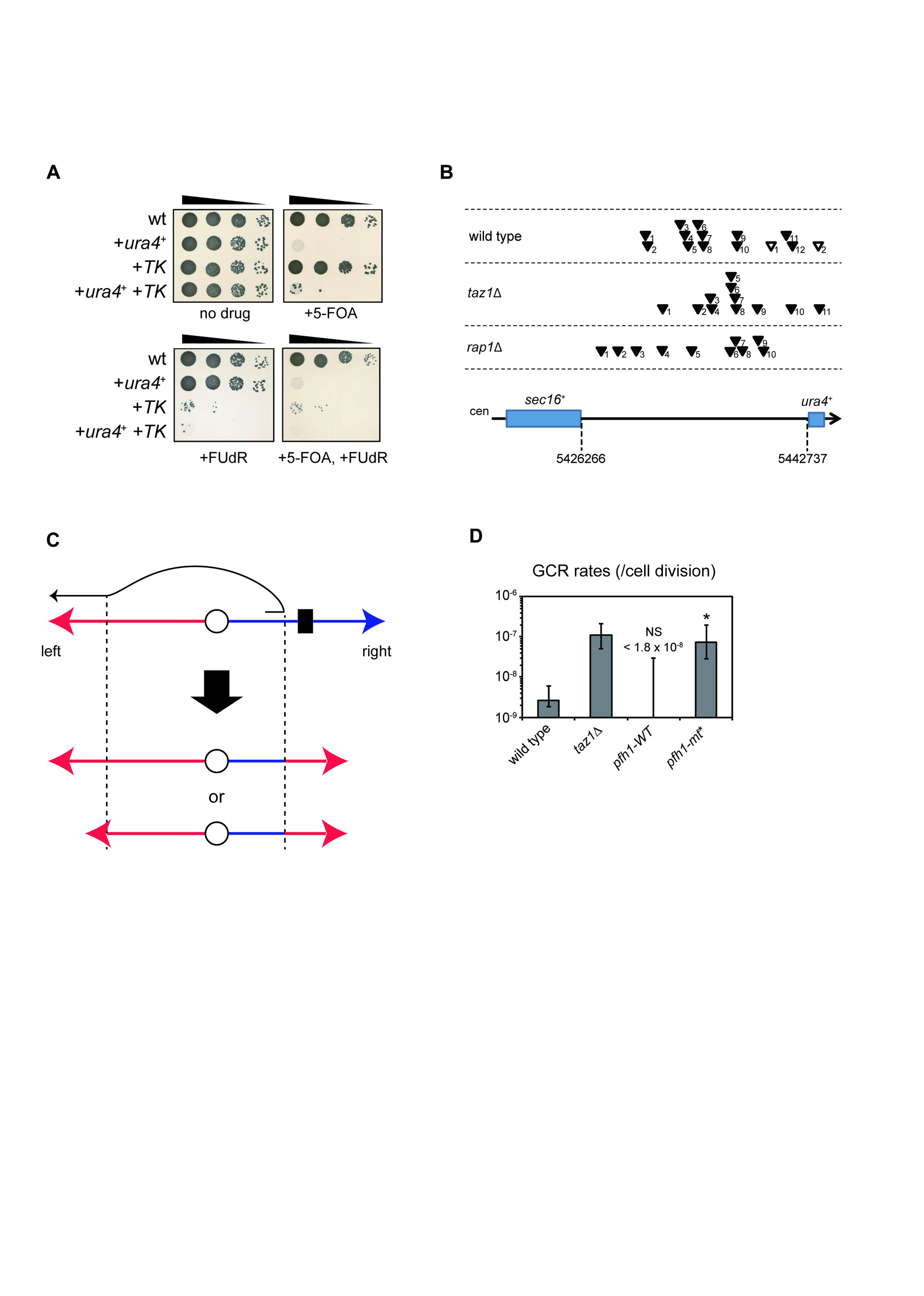

Supplement: S1 Fig — (A) Sensitivity of cells with ura4+ and/or TK to 5-FOA and/or FUdR. The wild-type strain in this experiment has neither ura4+ nor TK. Cells diluted by 1:10 serial dilutions were spotted on YES agar plates containing the indicated drugs and incubated at 32°C. (B) Locations of GCR junctions in the breakpoint region. Subscripts at arrowheads correspond to the numbers assigned to each GCR survivor in S2 Table. Nucleotide coordinates of the 3’ end of sec16+ and the 5’ end of inserted ura4+ are shown. Closed and open arrowheads indicate deletion and translocation types, respectively. (C) Schematic representation of the translocation observed in GCR survivors derived from wild-type cells. In these strains, the breakpoint region was joined to the chromosome 1 left arm in the opposite orientation. We have not addressed whether the original chromosome 1 left arm was retained or not. (D) GCR rates of strains expressing wild type or mutant Pfh1. Pfh1 constructs were expressed from original pfh1+ promoter integrated on genome. Asterisk and NS represent P<0.05 and P>0.05, respectively, relative to wild type. (TIF) [file pgen.1008335.s006.tif]

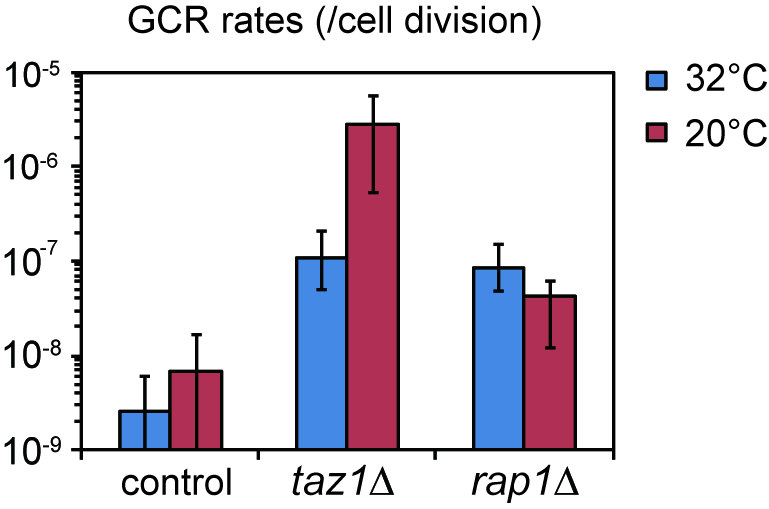

Supplement: S2 Fig — GCR rates of wild-type, taz1Δ, and rap1Δ strains incubated in 32°C and 20°C. (TIF) [file pgen.1008335.s007.tif]

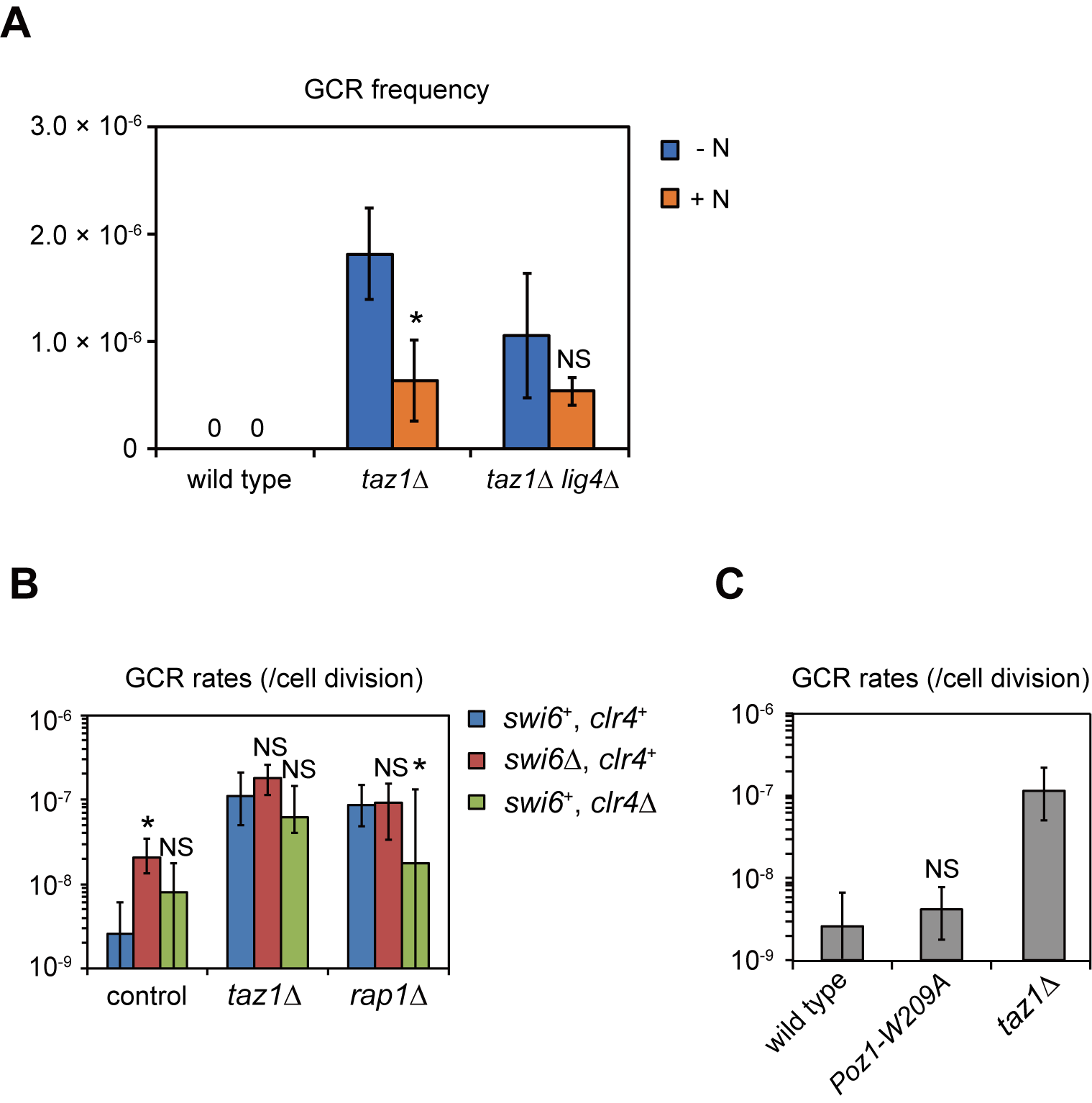

Supplement: S3 Fig — (A) GCR frequency of indicated strains in the presence and absence of nitrogen source in media. Please note that the GCR frequencies shown in this figure was measured by a different method from GCR rates shown in the other figures (see Methods). (B)(C) GCR rates of strains lacking essential factors for heterochromatin (B), and a poz1 point mutant that is defective in heterochromatin at telomeres (C). (TIF) [file pgen.1008335.s008.tif]

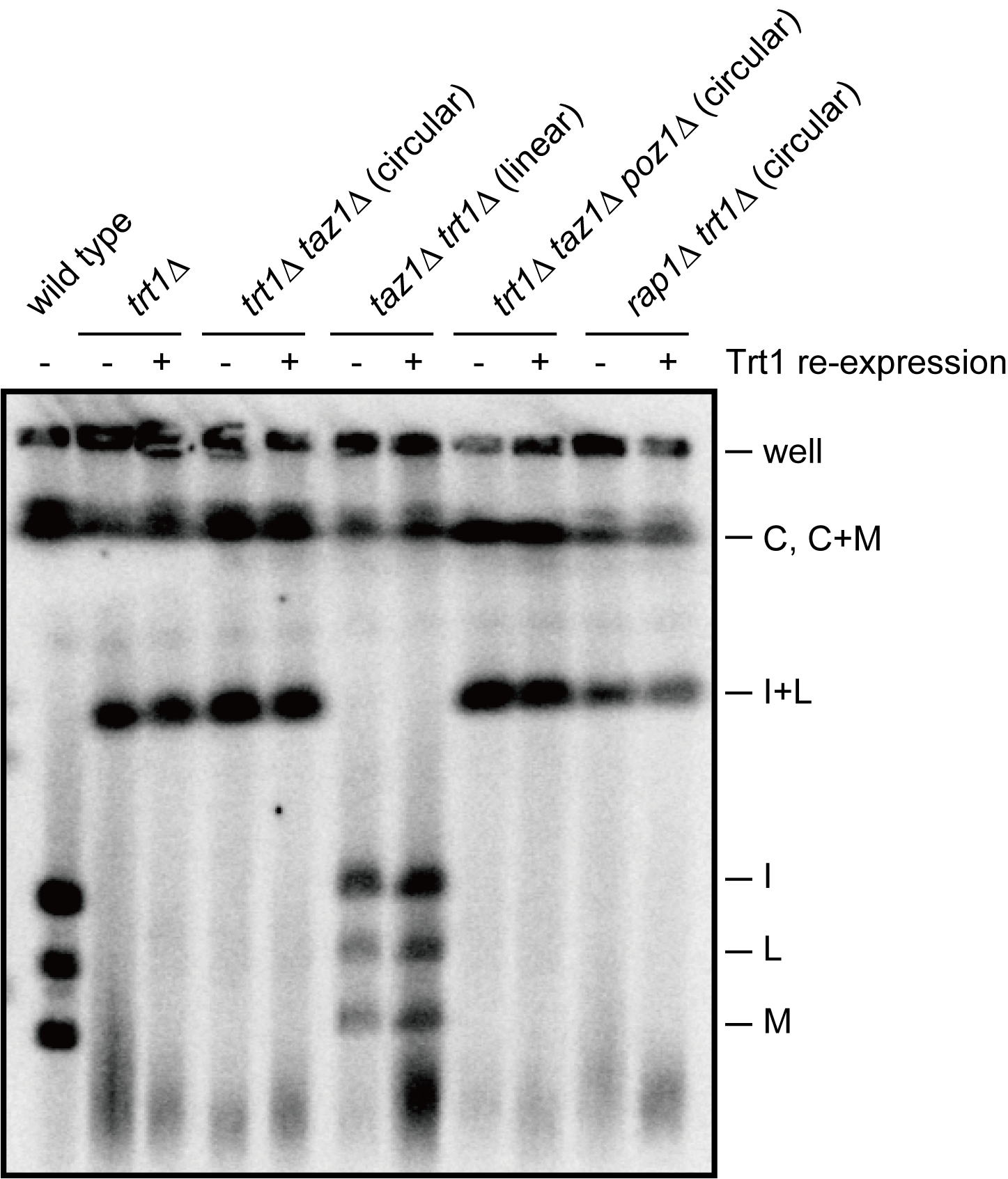

Supplement: S4 Fig — Maintenance of circularization of chromosome I & II in trt1Δ strains after Trt1 re-expression. NotI-digested chromosomes were analyzed by pulsed-field gel electrophoresis and Southern blotting. The terminal fragments of chromosome I and II were detected using a mixture of four probes detecting C, I, L, & M chromosome fragments. (TIF) [file pgen.1008335.s009.tif]

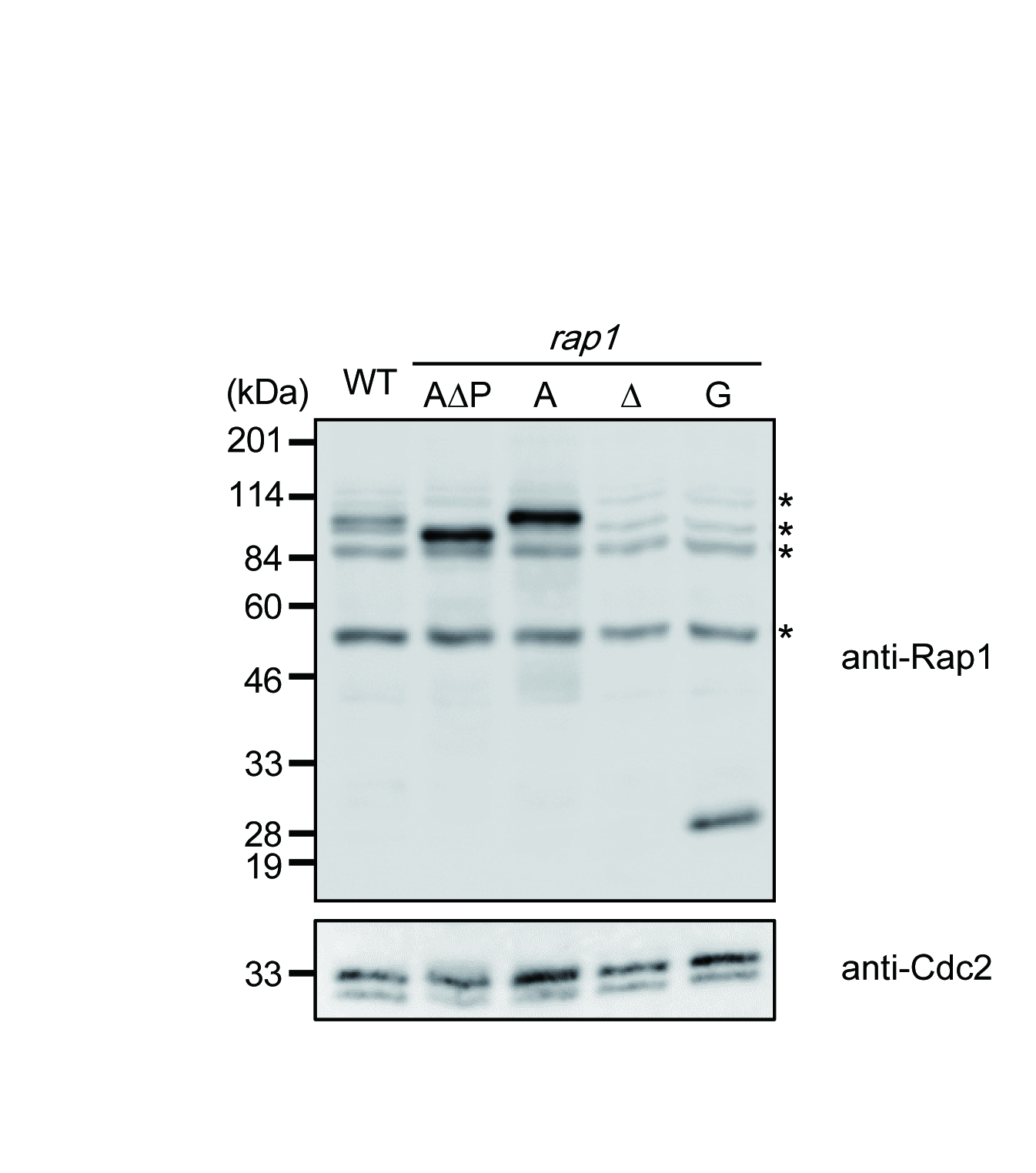

Supplement: S5 Fig — Expression of the Rap1 proteins. The whole cell extracts were analyzed by immunoblotting using anti-Rap1 antibody and anti-PSTAIR antibody for Cdc2 (loading control). WT, wild-type; and Δ, rap1Δ. Asterisks indicate non-specific bands. (TIF) [file pgen.1008335.s010.tif]

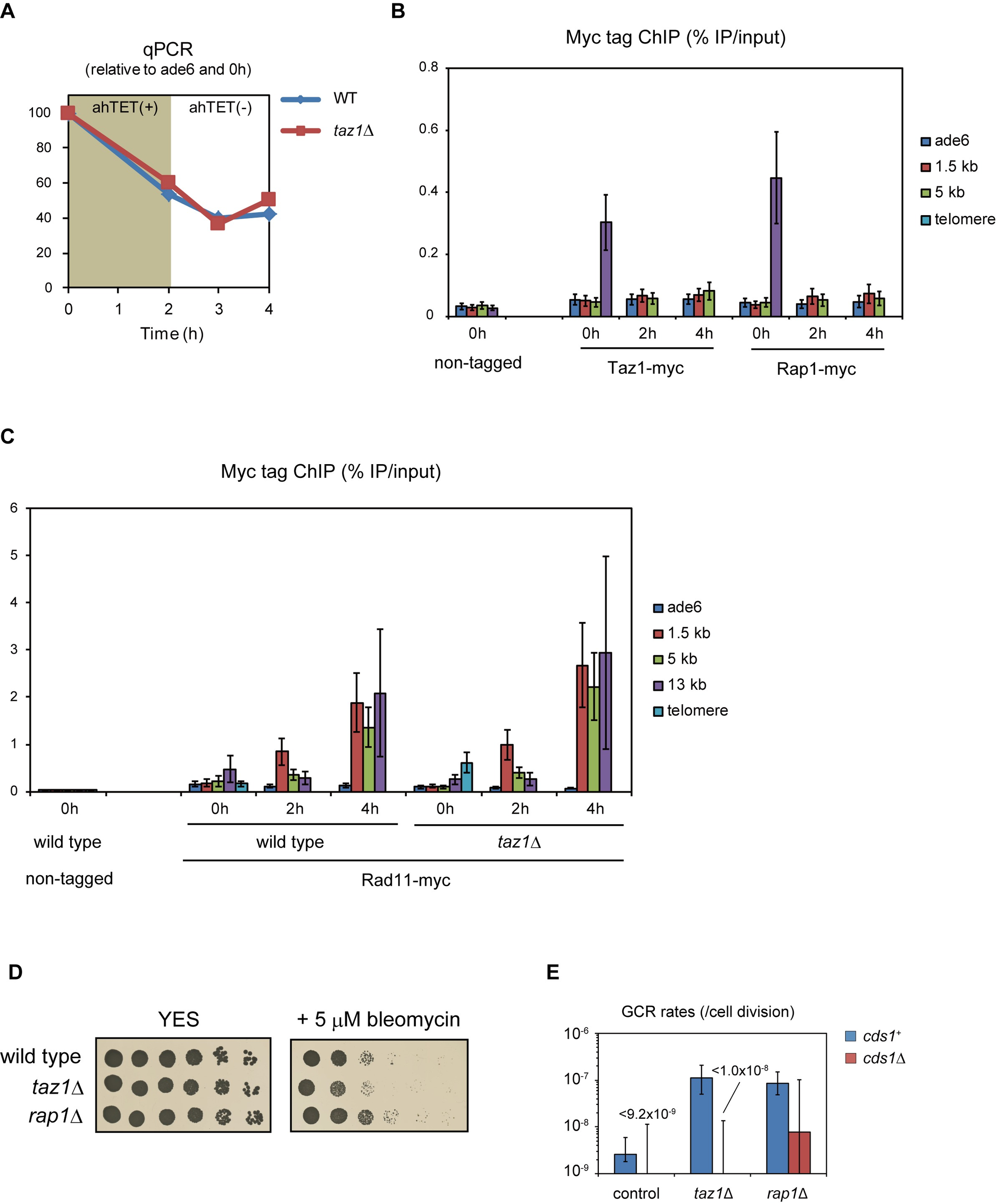

Supplement: S6 Fig — (A) Efficiency of DSB induction in I-SceIcs in an experiment conducted with the same protocol of Fig 6B, except that cells were washed out ahTET two hours after ahTET addition. N = 1. (B)(C) Localization of Taz1, Rap1, and Rad11 around I-SceI cut site. Cells expressing (B) Taz1-myc or Rap1-myc and (C) Rad11-myc were examined by ChIP using anti-myc antibody. Mean values ± SEM are indicated. N = 3. (D) Sensitivity of wild type, taz1Δ, and rap1Δ strains to bleomycin. Cells serially diluted by 1:5 were spotted on YES agar plates with indicated drug and incubated at 32°C. (E) GCR rates of wild-type, taz1Δ, and rap1Δ strains in the presence or absence of cds1+. (TIF) [file pgen.1008335.s011.tif]

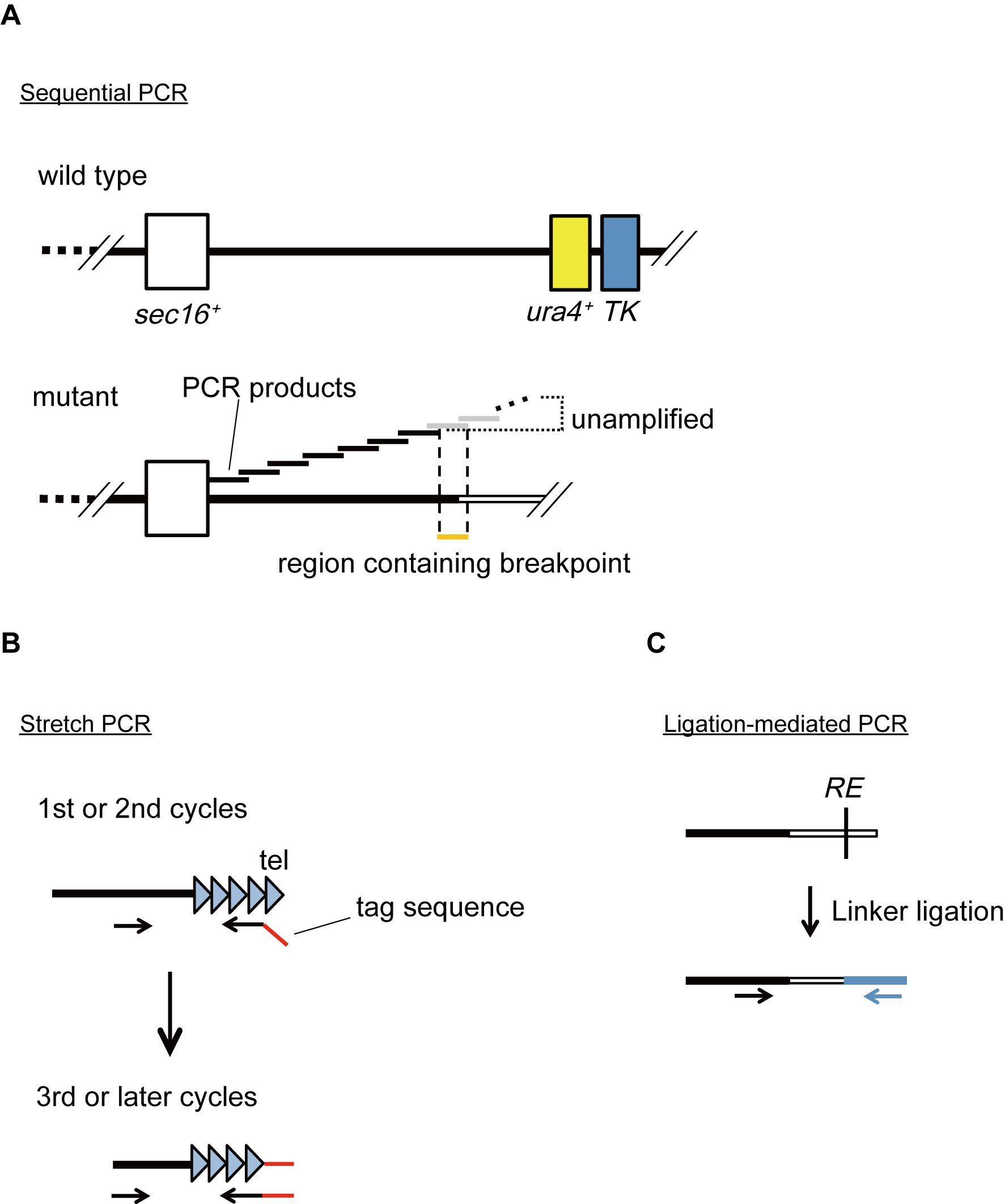

Supplement: S7 Fig — Schematic representation of the strategy for characterizing GCR breakpoints. (A) Sequential PCR to narrow down the location of breakpoints. Solid and empty thick lines represent original and newly added sequences, respectively. (B) Stretch PCR to assess telomere addition [61]. (C) Ligation-mediated PCR to assess translocation. See Materials and Methods for details. (TIF) [file pgen.1008335.s012.tif]

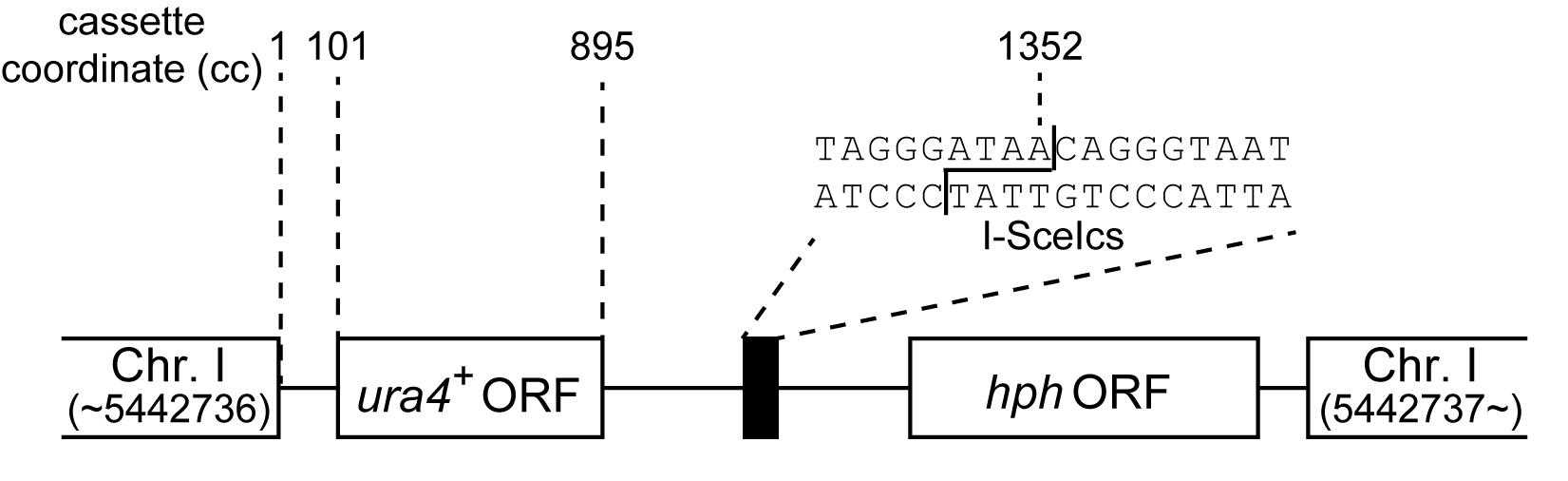

Supplement: S8 Fig — (TIF) [file pgen.1008335.s013.tif]
